# Fighting an Epidemic in Political Context: Thirty-Five Years of HIV/AIDS Policy Making in the United States

**DOI:** 10.1093/shm/hky108

**Published:** 2018-12-28

**Authors:** Tasleem J Padamsee

**Keywords:** HIV/AIDS, health policy, United States, policy history

## Abstract

The history of US government action on HIV/AIDS offers important lessons concerning the limits and possibilities of US public health policy. Yet only the first decade of this history has previously been well-documented. This article updates the history by constructing a macro-level account of policies that have been considered and implemented, along with the discourses and debates that have shaped them. This account is generated through systematic study of many dozens of policy making moments, drawing on >70 original interviews, >20,000 daily news reports and hundreds of contemporaneous policy documents. The paper chronicles HIV/AIDS policy from the initial years when the federal government resisted addressing the crisis; through subsequent periods shaped by alternating Republican and Democratic administrations; to contemporary policy making in an era when broader health policy transitions offer hope of normalized treatment and coverage for people with HIV, and scientific innovations offer the possibility of ending HIV/AIDS itself. It also illuminates how national HIV/AIDS policy is not only a series of responses to the concrete challenges of a health crisis, but also a malleable political product and a resource used to wage broader social and ideological battles.

## Introduction

The history of HIV/AIDS policy making offers important lessons for both scholars and practitioners, illuminating enduring patterns that may well continue to shape national policy choices in—and beyond—the HIV/AIDS arena. Thus far, however, only the first decade of US policy history has been well documented. A number of studies highlight individual debates and controversies from more recent years, but none attempts to characterise or explain the broader trajectory of AIDS-relevant policy making after 1990. This article helps fill the gap in our understanding by constructing a macro-level account of national policy debates and outcomes from 1981 to 2015. Encompassing the roles of community organisations, state and local governments, national and regional advocates, public health leaders and politicians, this account focuses on *what happened* in the arena of national HIV/AIDS policy, and *why*.

In discussing the well-documented period 1981–90, I rely largely on the excellent work of prior scholars and journalists, including Dennis Altman, Ronald Bayer, Jennifer Brier, Steven Epstein, David Kirp, Sandra Panem and Randy Shilts. To reconstruct the largely un-catalogued history of US HIV/AIDS policy making after 1990, I employ and triangulate three forms of data. The first comprises more than 20,000 news stories from *The New York Times* (*NYT*) and *Kaiser Daily HIV/AIDS Report*.^1^ Read in tandem with select legislative histories, the *NYT* and *Kaiser* reporting offers a detailed view of unfolding policy processes, including not only successful legislative proposals, but proposals that failed; proposals that never drew legislative attention; executive and regulatory actions; public controversies; and advocates’ attempts to raise issues to public attention.^2^ The second comprises more than 70 original, semi-structured interviews I conducted in 2005 and 2010–12 with policy makers and advocates who played key roles in the formulation of federal HIV/AIDS policy across various administrations (see Table 1 for a listing of informants’ roles).^3^ Policy documents produced by governmental and non-governmental stakeholders help to round out insights into policy making dynamics gleaned from the interviews.^4^ Thirdly and finally, the paper draws at select points on parallel data from the United Kingdom, including: news articles from *The Guardian*, *The London Times*, *The Financial Times*, and *Parliament Publications Database*; 55 semi-structured interviews I conducted with key policy makers and advocates who contributed to HIV/AIDS policy decisions in the UK; and British government and advocacy documents.^5^

**Table 1.** Policy making roles of US key informants

**Total informants**	**78**
Named	44
Confidential	34
**Direct Government service**	**60**
Executive advisory groups	18
Legislators and staff	13
Agency / department leaders	41
**Outside of Government**	**57**
Leaders of national policy advocacy groups	36
Leaders of consulting and research organizations	21

With over a million people living with HIV, the USA has the largest caseload among Western industrialised nations.^6^ The National Institutes of Health (NIH) spends over $3 billion each year on HIV/AIDS research, and many cutting-edge treatments and testing technologies are developed in the USA.^7^ With both a strong incentive to intervene and the technical capacity to do so, how has the US government responded? Over the past 35 years, policy results have varied by sector—from the relatively smooth facilitation of research on the causes, course and treatment of the disease; to the long, slow evolution of mechanisms to finance treatment for people with HIV; to repeated disagreements and policy reversals when it comes to prevention strategies.

A number of organising principles might have emerged from analysis of these policy trends. The state of research into HIV/AIDS prevention, testing and treatment could logically be expected to exert a strong guiding influence—and scientific developments do in fact impact policy deliberations. They are not determinative, however, as treatments are sometimes generated but not delivered, and prevention programmes are sometimes proven effective but not used. Similarly, the distribution and growth of the epidemic influences the course of policy debates without consistently determining outcomes: the needs of some highly-affected populations are addressed more promptly or consistently than others. Economic cycles exert their influence on policy as well, since discretionary programmes are often squeezed when federal budgets are tight. Yet the impact of economic factors is simultaneously constrained by policy: where there is no political will to deliver a service, or no mechanism in place to do so, the hypothetical availability of funds does not matter.

Instead, the clearest organising principle to emerge is the alternating cycle of Republican and Democratic presidencies. As the following account reflects, technological, epidemiological and fiscal factors interweave with political and ideological factors in determining outcomes; there is no mono-causal account of HIV/AIDS policy making to be had. However, the presidential administrations—with their contrasting social, economic and cultural worldviews, as espoused within the USA’s strongly entrenched two-party political tradition—emerge as the factor that most markedly shapes policy into discrete periods, influencing the reception of scientific data, the relative attention paid to various populations, and budget allotments. Accordingly, the periodisation of my account reflects the ascendance of the political and ideological in shaping national responses to HIV/AIDS.

## The Long Road to Action: 1981–1989

The earliest chapter of the American AIDS policy story is most often told as a tale of delayed and limited national leadership. Since this period has already been well documented and analysed, I will limit my treatment of it here. Nevertheless, a review of developments from 1981 to 1989 is crucial to understanding the epidemiological trajectory of HIV/AIDS, the growth in public awareness and fears, and the rise of HIV/AIDS interest groups and activism. This review sets the stage for understanding the dramatic way that new treatment regimens would affect both medical prognosis and popular conceptions of the disease. A review of the Reagan years is also vital to establishing the central role that presidential administrations (and their parties’ key constituencies) play in shaping discrete periods of national response to HIV/AIDS.

The pathogen that would come to be known as Human Immunodeficiency Virus (HIV) likely circulated for decades before attracting systematic attention.^8^ Urbanisation, globalisation, changing patterns of sexual contact and new distributions of other disease agents all contributed to rising transmission rates during the 1970s; by 1979, health professionals began to recognise an epidemic.^9^ In June 1981, the Centers for Disease Control and Prevention (CDC) published the first official US report on the new disease. Over the following months, AIDS took a swift and devastating toll on urban gay communities, with the hundreds of cases diagnosed that year representing only a tiny fraction of those already infected.^10^ However, the political climate deterred any proactive federal response. In keeping with his agenda of trimming federal government, the new president, Ronald Reagan, had cut budgets at the NIH and CDC. The social conservatives who helped elect him recoiled from a disease publicly associated with gay men—a strongly marginalised and stigmatised social group.^11^ Federal agency leaders expressed mounting frustrations over the next several years, as the administration blocked congressional appropriations for AIDS-related programmes and impeded the CDC’s attempts to mount prevention campaigns. Medical research at the NIH was relatively less affected by government recalcitrance, but for those already infected or at risk of contracting HIV little federal help was forthcoming. In 1983, activists succeeded in having AIDS designated a disability under Medicaid—but most AIDS patients died before receiving any benefits.^12^

As the federal response stalled, hard-hit communities mobilised. In the wake of the 1969 Stonewall Riots, gay community groups had formed in cities across the nation, often working to educate gay men about Hepatitis B and promote research on sexually transmitted diseases.^13^ Now they quickly retooled to provide support services to the ever-growing numbers of predominantly young men who were sick and dying from AIDS, and organisations such as New York City’s Gay Men’s Health Crisis, the Los Angeles Gay and Lesbian Center and San Francisco’s AIDS and KS (Kaposi’s Sarcoma) Foundation produced sex-positive, norm-based prevention programmes that curtailed infection rates among gay men within the first three years of the epidemic. Haemophiliac organisations mobilised quickly too, providing support services to patients infected through blood transfusions and lobbying the Public Health Service to protect the blood supply. Community organisation leaders also pushed for responses from their local governments, and some cities—most notably San Francisco—did take action by funding prevention education, support services and community-based research projects.^14^ It did not take long for leaders in these communities and cities to understand that local responses would not be equal to the task of confronting HIV/AIDS. Nevertheless, federal neglect persisted through to the end of 1985, when President Reagan finally acknowledged publicly the existence of AIDS—even as his administration proposed further cuts in AIDS spending.^15^

Mid-decade, two events awakened the possibility of federal leadership. In 1985, the death of actor Rock Hudson became the first high-profile celebrity death officially attributed to AIDS. This loss may have moved Reagan—a close personal friend of Hudson’s—to confront the mounting toll of HIV/AIDS on communities and health infrastructure; it certainly galvanised public concern.^16^ Then, in 1986, the Institute of Medicine (IOM)/National Academy of Sciences published a report noting the extreme fiscal strains mounting at hospitals in hard-hit cities and advocating a strong, coordinated response to the disease. Later that year, Reagan’s own Surgeon General, C. Everett Koop, followed suit. Both reports rejected the coercive and punitive measures being advocated by conservatives and recommended massive public education campaigns, increased availability of confidential testing, and a long-term, comprehensive research programme.^17^ The combination of heightened public concern and official policy recommendations made it increasingly difficult for the Reagan administration to continue its concerted policy of non-action. Activists were finally able to pressure President Reagan into appointing the Watkins Commission to investigate HIV/AIDS, and the country inched toward action. Still, the US government’s slow response and halting incrementalism contrasted sharply with events in Britain, where Margaret Thatcher’s Conservative government had already assumed proactive leadership on HIV/AIDS by this point.^18^

Toward the end of 1987—more than a year after the other major industrialised nations^19^—the US government took steps to raise AIDS awareness.^20^ The Department of Health and Human Services (HHS) mailed the Surgeon General’s findings to every American household, sponsored AIDS Awareness Month, and launched the ‘America Responds to AIDS’ advertising campaign.^21^ These high-profile actions offended many of the socially conservative congress-persons and advocates who held sway with the administration, but Surgeon General Koop and key congressional leaders, including Henry Waxman and Ted Weiss, ensured that HHS could proceed without political interference.^22^ Meanwhile, other agencies extended their activities. For instance, the Food and Drug Administration (FDA) worked on increasing condom effectiveness and visibility, while the CDC encouraged HIV testing and mandated state-level partner notification programmes.^23^ This was also the year that aerosolised pentamidine, or AZT, became available—a development that highlighted the treatment gap for low-income people with HIV. In a first attempt to address the gap, Congress made a direct allocation of $30 million to help states purchase AIDS drugs. Over the next few years, however, the problem would only become more pronounced as both HIV caseloads and prescription price tags rose, and efforts to address the treatment gap would remain patchwork until passage of the *Patient Protection and Affordable Care Act* (ACA) more than 20 years later.

The CDC also began making grants to community-based organisations (CBOs), in order to bolster prevention efforts among high-risk populations.^24^ Gay men’s health educators had already demonstrated the efficacy of candid, pro-sex approaches implemented at the community level, but social conservatives went on the attack, demanding and winning, *inter alia*, the 1988 Helms Amendment. This legislation required federally-funded programmes to emphasise abstinence and barred them from producing materials thought to ‘promote or encourage, directly or indirectly, homosexual sexual activities’.^25^ In place until 1992, the Helms Amendment forced most large, federally-funded CBOs to work directly contrary to public health expertise—for example by deleting pictures of genitals from their materials and omitting discussion of anal sex.^26^

In 1988 Congress also outlawed federal funding of needle exchange programmes, even as many other developed nations—from the Netherlands and the UK to Australia and New Zealand—moved to institutionalise needle exchange as a core prevention strategy.^27^ Comparison to the parallel policy making moment in the UK is particularly instructive here, since the USA and UK were similarly situated with respect to the problem of HIV transmission among urban injecting drug users; scientific and clinical discourses strongly favouring needle exchange as a harm reduction strategy; moral and political discourses from social conservatives who strongly objected to the strategy; and institutional capacities and mechanisms for launching needle exchange programmes.^28^ Yet their policy outcomes in 1988 were dramatically different.^29^ In the UK, testimony from epidemiologists, researchers, activists and clinicians led Scotland’s McClelland Committee (formed in response to Edinburgh’s alarmingly high transmission rates) to conclude in late 1986 that instituting needle exchanges made sense, once the risks drug users faced were considered on a ‘hierarchy of needs’. In response, the UK’s Department of Health and Social Security initiated pilot needle exchanges.^30^ The Home Office favoured tougher law enforcement against drug users and mounted fierce resistance from within the Thatcher Administration.^31^ However, when the pilot programmes showed that needle exchanges could reduce HIV transmission, cementing the scientific consensus concerning their efficacy, conservative critics were effectively silenced. Nationally-funded needle exchange programmes were permanently authorised in 1988.^32^ US politicians, by contrast, did not view the scientific evidence as locking them into a course of action.^33^ HHS Secretary Louis Sullivan was interested in the harm-reduction potential of needle exchange, but Director of the Office of National Drug Control Policy William Bennett pushed more effectively against harm reduction.^34^ In Congress, expert testimony in favour of needle exchange was effectively countered by social conservatives, who argued the approach would legitimise illegal drug use. In 1988 legislators passed a unilateral ban on federal funding of needle exchange programmes—a decision that shapes US policy on injecting drug use to this day. Although it is difficult to demonstrate a definitive causal relationship, researchers generally agree that national needle exchange programmes have helped reduce infection rates among injecting drug users. In the UK the infection rate for this population is less than 3 per cent; in the US this rate is upwards of 15 per cent.^35^

As George H. W. Bush assumed the presidency at the start of 1989, HIV/AIDS activism heated up, driving changes in policy arenas beyond prevention. ACT-UP’s Treatment Action Committee organised direct confrontations with FDA and NIH officials, winning dramatic regulatory changes that widened access to experimental treatments and accelerated drug approval processes by two or more years.^36^ Activists convinced federal agencies to include patient representatives and lay experts in their decision making bodies, and they began other efforts—such as pushing the CDC to recognise the symptomology of AIDS in women and IV-drug users—that would yield dividends in later administrations.^37^ Outside the treatment arena, rights both expanded and contracted: discrimination against HIV-positive citizens became illegal, while new immigration policies prohibited foreigners with HIV from entering the country.^38^

As of 1990, death tolls from AIDS continued to mount, but the confusing newness of the disease was passing. Public interest was keen; activism was intense; and the federal government had begun to act in the areas of prevention, treatment and research.^39^ As the second presidential administration to confront the epidemic, the Bush White House did not assume a leadership role on HIV/AIDS policy, but was less obstructionist than its predecessor.^40^ Hence, by the end of the 1980s the stage was set for a more proactive federal response. It is also at this point that most existing histories of US HIV/AIDS policy end—and here that the paper’s original analysis begins.

## Turning Points: 1990–1992

The passage of the Ryan White Comprehensive AIDS Resources Emergency (CARE) Act in 1990 opened the next major chapter of the American HIV/AIDS policy story, which saw the transition from a nascent, largely reactive national response to a more forward-looking approach institutionalised broadly across the federal government. The CARE Act aimed to address the critical problem of financing treatment for HIV/AIDS patients, a significant proportion of whom were under- or uninsured, particularly as HIV became increasingly concentrated in poorer communities. Employment-based private insurance, the cornerstone of the US health care system, was failing people with HIV. Private insurance companies dumped HIV-positive patients from their rolls or used ‘catastrophic illness’ exemptions to refuse to cover AIDS-related costs.^41^ Many other patients lost coverage when they became too sick to work. Medicaid quickly became the largest single payer for HIV/AIDS treatment in the nation. Individuals with HIV/AIDS had to qualify for Medicaid, however, in one of three specific ways: they were extremely poor prior to their illness; they ‘spent down’ all their resources after becoming sick, *until* they were extremely poor; or they survived the two-year waiting period after diagnosis of full-blown AIDS, allowing them to receive Medicaid.^42^ The traditional combination of private insurance and Medicaid thus left significant gaps, not only in who was covered but in the periods of illness for which they were covered.^43^

Hospital care for the US uninsured has traditionally been financed through cost-shifting mechanisms and charitable donations, but by 1990 it was clear these were wholly inadequate for addressing AIDS, particularly in urban centres. In the mid-1980s, policy advocates had pushed for a federal response to the treatment financing problem, and various legislative fixes had been proposed—and failed—in Congress.^44^ Now, however, the fiscal strain began to jeopardise the availability and standard of care for people *without* HIV, as long-solvent urban hospitals went bankrupt under the strain of treating uninsured AIDS patients and hospital closures loomed.^45^ This fact initiated a crucial shift in political discourse, and AIDS treatment financing came to be understood as a general problem that the US government should address.^46^

Beyond the fiscal crisis and the newly emerging discourse of generalised risk it engendered, two other factors enabled passage of the CARE Act. First, breakthroughs in medical treatment shifted the issue of AIDS treatment financing from one focused on palliative and supportive care to a more compelling one about effective treatments that could save lives.^47^ Second, policy advocates distanced the bill from gay men and drug users by marshalling and reenergising a pre-existing discourse of AIDS’ ‘innocent victims’—a move that helped build public and congressional support. The name of the legislation reflects this strategy: Ryan White was an Indiana teenager who contracted HIV during routine treatment for haemophilia and died of AIDS in 1990.

In response to these conditions, Congress passed the CARE Act in 1990, and President Bush signed it into law.^48^ The CARE Act changed the landscape of US HIV services by establishing new agencies, organisations and funding structures that allowed localities to provide health care and prescription drugs, as well as transportation, case management and other supportive services, to needy HIV patients. In policy terms, the CARE Act both reflects the devastating slowness of the US response to HIV/AIDS and represents a crucial policy innovation. With respect to the former, US attempts to address the treatment financing gap lagged a full three years behind the UK response (and in the context of a deadly epidemic still on the rise, three years represents a significant human toll).^49^ With respect to the latter, when the US did finally address the issue through passage of the CARE Act, the country created a rare safety net under both private and public insurance systems.^50^ In a national health system where income largely determines access to health care, people with HIV/AIDS became one of the few American groups for whom this was not the case.^51^

Two additional developments during the period 1990–92 helped transform life for Americans with AIDS. First, AIDS patients gained protection under the 1990 Americans with Disabilities Act (ADA), which not only prohibits discrimination but requires government and businesses to make ‘reasonable accommodations’ to allow disabled individuals to work, travel and shop.^52^ Second, in 1991 Congress quietly authorised the Housing Opportunities for People with AIDS Act (HOPWA).^53^ Despite the lack of fanfare surrounding its passage, HOPWA—which received notable funding increases during the Clinton administration—became a critical programme for those with HIV/AIDS, and helped demonstrate the power of housing programmes to improve their health outcomes.^54^

During the brief, transitional period of 1990–92, the ideas and impetus for key HIV/AIDS policy decisions continued to originate largely outside federal government and represented the fruits of a decade’s worth of investigation and activism. Adopted primarily through Congressional action, these changes were possible in the context of a presidential administration that, while still largely passive on HIV/AIDS, was less committed to the hard-line social and economic conservatism that made action on the crisis so difficult under President Reagan. Beyond seeking to protect the uninfected population, the federal government now acknowledged a specific responsibility to address the needs of people with HIV/AIDS.

## Leadership and Resistance in the Clinton Years: 1993–2000

In 1993, William Jefferson Clinton became the first Democratic president to confront the HIV/AIDS epidemic. The Clinton campaign had pledged a stronger response on AIDS, and experienced policy advocates hoped his administration would galvanise a systematic and aggressive federal approach.^55^ The next eight years would, indeed, witness more proactive executive leadership, as well as the rapid expansion of federal HIV/AIDS programme budgets. Yet policy making during the Clinton years would also be constrained by competing political demands from the Left and the Right, particularly after the 1994 mid-term elections.

Early on, Clinton created two administrative mechanisms for bringing HIV/AIDS experts and advocates into closer contact with policy makers. First, in fulfilment of a campaign promise to activists, Clinton created the role of National AIDS Policy Coordinator. The nation’s first ‘AIDS Czar’, Kristine Gebbie, was a former state health commissioner who lacked the resources and insider connections needed to drive significant policy change and resigned within a year.^56^ However, the post gained influence with the 1997 appointment of Sandra Thurman, a former AIDS service organisation director, who forcefully pushed the White House AIDS policy agenda throughout Clinton’s second term.^57^ Second, in 1995, Clinton created the Presidential Advisory Council on HIV/AIDS (PACHA), a panel of science and policy experts empowered to conduct research; hold hearings; issue official positions and recommendations; exert pressure on Congress and federal agencies; and publish yearly reviews (often quite critical) of the nation’s progress on AIDS.^58^ In addition to these administrative innovations, from 1995 to 2001 Clinton oversaw yearly increases of between 9.7 and 15.5 per cent in federal spending on HIV/AIDS, for a total increase of 73 per cent, or $4.87 billion—an achievement for which HHS Secretary Donna Shalala shares credit.^59^

Policy debates during these years focused most frequently on access to medical treatment, an issue that gained new urgency in 1996 with the advent of protease inhibitors and Highly Active Anti-Retroviral Treatment (HAART), a long-term cocktail of (typically) three drugs that work against HIV in various ways. The early HAART regimen was demanding—involving many pills taken on a precise schedule—and could produce dangerous side effects and complications, including drug-resistant viral infections. Nevertheless, HAART transformed the experience of life with HIV, as thousands of patients on the verge of death in early 1996 experienced a ‘Lazarus Effect’, and AIDS death rates began to drop precipitously.^60^ HAART also transformed the policy landscape, since the availability of effective long-term treatment both reinforced ethical arguments for treatment access and changed the calculus of public costs. Experts had long agreed that early treatment offered significant medical benefits; now they argued for its societal benefits as well. Even factoring in longer life expectancies, HAART cost less than treating recurrent, life-threatening emergencies in patients without access to it. Moreover, drug therapy reduced transmissibility, and fewer transmissions meant lower future costs.^61^

In the short term, however, HAART was expensive. Meanwhile, the CARE Act—which was meant to fill the cracks of America’s patchwork insurance system for people with HIV—was chronically under-funded and was, as a discretionary programme, subject to reauthorisation every five years, more or less ensuring cyclical congressional debate. One high-profile reauthorisation controversy from 1995 concerned the issue of mandatory testing of pregnant women and newborns. The issue pitted conservative proponents of mandatory testing—led by Congressmen Jesse Helms, Bob Dole and Newt Gingrich—against AIDS advocates, who feared the approach would deter women from seeking medical care.^62^ The compromise bill that President Clinton eventually signed in 1996 provided for the development of guidelines for testing pregnant women (although work on the guidelines would not proceed until 1998, when the IOM called for routine prenatal testing).^63^ The 2000 reauthorisation debates centred on funding for localities, pitting legislators from regions that were hit by the epidemic early on and now had well-established programmes to protect against legislators from regions newly struggling to respond. Again the result was a compromise: more dollars were channelled to emerging epidemic centres, while ‘hold harmless’ provisions limited the amount localities like San Francisco could lose in any given funding year.^64^ Across multiple reauthorisations, it should be noted, one area of the CARE Act consistently expanded: the AIDS Drug Assistance Programs (ADAPs), which funnelled federal dollars to help state governments help purchase HIV drugs for people who could not otherwise afford them.^65^ Even so, ADAP resources consistently fell short of demand, forcing patients in states with less generous ADAPs to forgo prescribed medications—or leaving them to die while on ADAP waitlists.^66^ The need to ensure access to long-term treatment for everyone with HIV thus exacerbated a crucial policy problem.

During the Clinton years, several incremental measures prevented the erosion of treatment access and helped to keep HIV-positive individuals insured. The poorest HIV-positive Americans retained Medicaid coverage, as Democrats resisted (and sometimes reversed) yearly, Republican-sponsored attempts to trim the programme.^67^ In 1993, new disability rules made it easier for HIV-positive individuals to qualify for Medicare, and the 1999 Ticket to Work Incentives Improvement Act allowed those HIV patients able to return to work to stay on Medicare longer.^68^ However, the larger goal of insuring all people with HIV proved unattainable. The Early Treatment of HIV Act (ETHA)—developed by HIV/AIDS advocates and introduced in several Congresses starting in 1999—would have extended Medicaid categorically to all people living with HIV.^69^ The Clinton Administration, PACHA, and numerous health care experts backed ETHA, as did a growing number of congress-people, but Republican control of Congress made it impossible to pass.^70^

While treatment issues took centre stage during the Clinton years, consequential policy developments were also underway in the research arena. The 1993 NIH Revitalization Act empowered the Office of AIDS Research (OAR), established in 1988 as a coordinating body, to set priorities and budgets for all HIV-related research across the usually-autonomous federal research institutes.^71^ This innovation has proven both controversial and resilient: after years of debate over OAR’s role, HIV/AIDS remains the only disease for which US government-funded research is coordinated in this manner.^72^ Additionally, in 1997, Clinton established the Vaccine Research Center (VRC), charged with developing an HIV vaccine within 10 years. Vaccine research was also bolstered by the 2000 Millennium Vaccine Initiative, which incentivised pharmaceutical companies to pursue a vaccine.^73^ Although a vaccine has proved elusive, Clinton initiatives helped make it a major focus of scientific effort.

Prevention policy changed as well. For instance, in a quiet move that would have profound effects, the CDC instituted a new ‘community planning’ process that allowed community grantees to make local decisions in allocating their federal prevention funds, provided they employed evidence-based interventions and met inclusion, representation, and parity requirements.^74^ Not all prevention policy making was so smooth, however, as needle exchange once again became a hotly-debated, partisan issue, exemplifying the push-and-pull dynamic of these years.

When Congress banned federal funding of needle exchange programmes in 1988, it also stipulated—in a concession to public health experts—that the Secretary of HHS could rescind the ban if future research found such programmes (a) reduced the spread of HIV, and (b) did not increase rates of drug abuse. By 1995, as injecting drug use continued to be a major driver of the US epidemic, a panel of National Research Council and IOM experts concluded that both criteria for lifting the ban had been met.^75^ Far from stimulating a simple policy reversal, however, their conclusions initiated four years of political contest. The weight of scientific evidence was with proponents for lifting the ban, including prominent AIDS activists, the Surgeon General, CDC leaders, congressional Democrats, PACHA and Clinton’s AIDS Czar Thurman.^76^ Among opponents were Clinton’s Drug Czar General Barry R. McCaffrey and socially conservative legislators. Although proponents of needle exchange marshalled convincing evidence that the approach could save lives and even reduce drug use, their opponents again won the day with moral arguments that needle exchange was antithetical to the ‘War on Drugs’. Ultimately, Secretary Shalala announced that needle exchange programmes had been proven effective, but that the ban would nonetheless remain in place.^77^ For years after this paradoxical decision, HIV/AIDS advocacy groups pushed the issue with Congress and federal agencies, congressional debate continued, and states and cities used their own (and private) funds to establish local programmes—but federal policy remained unchanged.^78^

A final area of policy activity during the 1990s involved recognition of the toll that HIV/AIDS was taking on racial minorities and women—populations that previously had received little attention. Since early in the epidemic, racial minorities had been disproportionately affected, but governmental response had lagged.^79^ Over 1991–93, the National Commission on AIDS began to investigate the racial dimension of AIDS, and the NIH pushed for minority inclusion in clinical trials.^80^ Public recognition of the issue also began to increase when African American basketball star Magic Johnson announced his positive HIV status in 1991.^81^ But larger-scale responses did not emerge until after a dramatic political episode in early 1998: African American community and health leaders attending a CDC briefing expressed outrage over the impact on racial minorities (who by then represented more than 40 per cent of new cases diagnosed annually) and demanded sweeping interventions. Some even walked out of the briefing in protest.^82^ In the flurry of activity that followed, black congressional leaders declared a ‘state of emergency’ and convinced the CDC to follow suit. That fall, the Congressional Black Caucus and AIDS advocates spearheaded a successful campaign to authorise the Minority AIDS Initiative (MAI): a new set of appropriations for treatment and prevention infrastructures within minority communities.^83^ MAI-related programmes and allocations have grown since then but not sufficiently to halt the dramatic growth of HIV among minorities, and the ‘epidemic of colour’ remains a central policy challenge.^,^84^^

The problem of HIV among women was also pushed to the fore. Although it had been clear since the early 1980s that women contracted HIV through heterosexual sex and other transmission routes, women systematically had been elided in both scientific and popular AIDS discourses, as well as in policy responses.^85^ Often, women were considered only as HIV vectors or ‘pass-throughs’ and blamed for the infection of men and children.^86^ Prostitutes were a particular focus of blame, despite the distinct lack of evidence that prostitution had much to do with the spread of HIV in Western nations.^87^ Infected mothers in ‘AIDS Baby’ cases likewise received condemnation but little policy help; even mandatory HIV testing conversations ignored women’s education and treatment, focusing solely on their babies’ future health.^88^ Beyond discourses of blame, the medical press, popular media, and mainstream feminist sources generally presented the view that ‘normal’ women did not get HIV—an assertion that dismissed ever-growing numbers of poor, African American, Latina and/or drug-using women with HIV as ‘abnormal’.^89^ Meanwhile, the very categories the CDC used to classify new HIV cases made it nearly impossible to document or assess the real HIV risks women faced.^90^

In the late 1980s, feminist scholars, activists (such as those of the ACT-UP Women’s Caucus) and policy advocates (including the Center for Women Policy Studies) began to mount vocal critiques of these trends.^91^ By the early 1990s their arguments gained traction. Particularly crucial was the issue of definition: an official AIDS diagnosis was needed to qualify for most federal assistance programmes, yet the existing clinical definition omitted important manifestations characteristic of groups other than gay men. Expanding the definition was nonetheless politically difficult, because the change would increase diagnoses by up to 100 per cent, thereby driving up federal expenditures. In 1993 advocates won out, and the CDC adopted a new definition of AIDS that included invasive cervical cancer, recurrent pulmonary tuberculosis and other conditions specific to women or injecting drug users.^92^ Also in 1993 the NIH launched the first study of the natural history of HIV in women, and in 1994 it began requiring grant applicants to address potential inclusion of women and minorities in their research.^93^ Despite these steps, efforts to tailor prevention and care models to women’s needs have lagged.^94^ Transmission rates among women have continued to rise—particularly as part of the growth in heterosexually-acquired infections within minority populations.^95^

## A Conservative Turn in the Bush Years: 2001–2008

If AIDS policy was hammered out by warring political forces under President Clinton, conservative forces took firm control under President George W. Bush. Changes to PACHA offer a snapshot of the broader transition: Bush appointed a number of prominent advocates of abstinence-only education who had little AIDS-related experience to the Council, and PACHA was transformed from an outspoken advocacy body to one that quietly supported White House policy directives.^96^ The Bush Administration also managed to reframe the AIDS issue—from a primarily domestic to a primarily international one—by initiating the President’s Emergency Plan for AIDS Relief (PEPFAR). PEPFAR poured millions of dollars into delivering medication to parts of the world where it would not otherwise have been accessible.^97^ Bush thus staked out leadership on the global pandemic with what he termed a ‘compassionate conservative’ approach to AIDS.^98^

Domestically, meanwhile, a profound shift was underway. This was particularly evident in the area of prevention policy, which was overhauled in line with the religious principles that had long organised conservative thinking on HIV/AIDS (e.g. abstinence from non-marital sex and condemnation of homosexuality).^99^ Within its first months, the Bush Administration began actively promoting abstinence-only education, in direct opposition to recommendations by the Surgeon General and despite objections that the strategy had never been proven effective and could heighten risks for youth.^100^ The CDC was directed to prioritise abstinence messages across the board, and over the next several years, an increasing proportion of prevention funding was channelled into abstinence-based programmes.^101^ Condom information was removed from federal government websites.^102^ The Administration lobbied—unsuccessfully—for condom labelling that would warn users of condoms’ limited effectiveness in protecting against sexually transmitted diseases.^103^ In 2003 HHS began requiring all foreign non-governmental organisations that received US HIV/AIDS prevention funds to adopt an ‘anti-prostitution pledge’—and in 2005 it extended this requirement to US organisations, over objections that prostitutes would be deterred from accessing critical prevention services.

Perhaps most emblematic of the determination to re-make HIV prevention policy was the push by high-ranking congressional Republicans, starting in 1999, to audit all federally-funded HIV/AIDS programmes.^104^ The most high-profile of these audits involved San Francisco’s Stop AIDS Project, whose workshops were dubbed ‘sexually provocative’ and ‘evil’ by prominent conservatives.^105^ After more than two years of investigation, the CDC concluded that the workshops reflected appropriate public health practice, grounded in scientific knowledge about HIV prevention and behaviour change. Nevertheless, much like the 1988 Helms Amendment, the audits prompted the CDC to tighten grant guidelines and pushed HIV/AIDS educators away from candid, comprehensive content toward vaguer, less community-specific messages.^106^ Federal audits extended from the prevention arena and into medical research, beginning in 2003, when congressional leaders audited the topics of NIH-funded projects and threatened to re-evaluate several grants related to HIV/AIDS, sexuality and risk behaviour. In response, academics mobilised through national organisations such as the Union of Concerned Scientists and Research!America to protect scientific autonomy and the peer-review process.^107^

Alongside the movement to better align HIV prevention with conservative values came other shifts in prevention, many of them geared toward routinising testing and the identification of HIV-positive individuals. In 2001, the CDC launched the Serostatus Approach to Fighting the HIV Epidemic (SAFE), a programme built around the common-sense principle that, since only HIV-positive individuals can infect others, it is their behaviour that matters most. With SAFE, the CDC moved away from the longstanding strategy of balancing general AIDS awareness programmes with ones targeted to high-risk groups; the CDC now focused on raising the number of HIV-positive people who knew their status, understood it, and received treatment for it, making them less likely to transmit the virus to others.^108^ In a 2003 guidance, the CDC advised that physicians should test all pregnant women for HIV unless they ‘opt out’, reflecting an attempt to make HIV testing a regular part of obstetric care. In 2006 the agency dropped longstanding requirements that every HIV test be accompanied by written consent and pre-test counselling. It also recommended that adults and adolescents in all health care settings be tested for HIV at least once and that those engaging in risk behaviours be tested annually.^109^ These routine testing policies sparked resistance from physicians and HIV/AIDS advocates, particularly in states where the epidemic had hit earliest and hardest, leaving affected communities with a vivid understanding of the discrimination and stigma HIV-positive individuals could face. Even though CDC leaders had determined that routine testing would benefit both those with HIV (by setting them on a road to effective treatment) and those without HIV (by clarifying the HIV status of partners or potential partners), Left-leaning activists worried about the potential for coercive testing of marginalised people and the stigma that those who tested positive might face.^110^ Conservative leaders were more focused on the broad societal benefits and less concerned about protecting the individual’s choice not to test. Despite opposition, the routine testing policies of the George W. Bush years initiated a radical shift in medical practice toward treating HIV as ‘just another medical condition’.^111^ That shift has continued to gain momentum in both discourse and institutional practice—for example through a 2013 US Preventive Services Task Force decision that yielded increased insurance coverage for HIV testing.^112^

Outside the prevention arena, HIV/AIDS policy making in the Bush years reflected similar themes in less dramatic form. Funding of all domestic HIV/AIDS programmes remained generally flat across the period, despite a still-growing epidemic, and attempts to provide government-funded health care for needy people with HIV/AIDS met even greater obstacles than during the push-and-pull Clinton era. Political struggles over Medicare resulted in repeated, temporary losses of prescription drug access for patients. Financial constraints on Medicaid, which covered many more HIV-positive people than Medicare, forced patients to forgo certain medications.^113^ This problem was exacerbated by pharmaceutical contracts that charged Medicaid up to 30 per cent more than other federal programmes for the same drugs.^114^ The state ADAPs alone saw funding increases; still they remained underfunded, serving fewer people than regulations allowed, maintaining waiting lists, and limiting their formularies to cope with high drug costs.

Finally, the CARE Act was changed substantially during the course of its third reauthorisation. Although delayed by Hurricane Katrina and war in Iraq, the 2006 reauthorisation process was long and, once again, contentious.^115^ With a Republican Congress and presidency, conservative views gained new traction on two long-standing controversies: geographical funding distributions and local control. With regard to the first, the final legislation increased proportional allocations to rural and Southern states, while still limiting to 5 per cent the amount that early epidemic centres could lose. With regard to the second, the approved legislation reduced local control over spending and curtailed support services through a new requirement that 75 per cent of CARE Act funds go to ‘core’ medical services, such as physician visits and medication.

## Reversals and Reforms under the Obama Administration: 2009–2016

Barack Obama’s presidency brought another swing of the HIV policy pendulum: renewed attention to the domestic epidemic, less restrictive prevention approaches, and concerted focus on expanding treatment. President Obama’s first HIV/AIDS policy steps in 2009 were ones that could be taken easily and definitively, given Democratic majorities in both houses of Congress. Building on one of Bush’s unfinished initiatives, Obama lifted the Reagan-era ban on immigration of HIV-positive people.^116^ The CARE Act was rapidly reauthorised and signed into law, and the administration held public meetings to bring attention to the HIV/AIDS epidemic among minority groups.^117^

The abstinence-based policies of the 2000s were largely dismantled. In 2009–10, the president and Congress increased overall expenditures on sex education by nearly $190 million but slashed funding for abstinence-only programmes by two-thirds, instead favouring evidence-based, comprehensive sex education initiatives for youth.^118^ CDC materials once again highlighted the role of condoms in HIV prevention, and the agency resumed dissemination of research on effective prevention strategies.^119^ It took no concrete steps to drop ‘anti-prostitution pledge’ requirements—perhaps reflecting the ongoing power of socially conservative discourses. However, HIV/AIDS activists continued to challenge the policy in court, arguing the pledges could prevent prostitutes—an important risk group—from accessing prevention services, thereby undermining efforts to control the epidemic. Several judicial decisions weakened the federal policy, and the Supreme Court overturned the entire law in 2013.^120^

These years also witnessed the next chapter in needle-exchange controversies. Seizing a rare moment when Democrats controlled Congress and Republicans were occupied by other issues, the Obama Administration quietly lifted the federal funding ban at the end of 2009.^121^ At the same time, the CDC generated guidelines for states wishing to fund needle exchange programmes with existing grants (although it did not explicitly encourage such uses).^122^ In 2012, after a short debate in which familiar themes were voiced by both sides, Congress reinstated the ban.^123^ However, needle exchange programmes continued to open and operate without federal funding around the nation, fuelled by both local need and a growing, global discourse critical of the ‘War on Drugs’.^124^ In 2015, as media outlets and public health experts described a national opioid epidemic, the door to federally-funded needle exchange opened once more, and the Obama Administration announced a bipartisan agreement allowing high-risk communities to use federal funds for syringe service programmes.^125^

The Obama administration also moved in new directions. Under Director Jeffrey Crowley, the White House Office of National AIDS Policy (ONAP) engaged a wide range of stakeholders to construct a comprehensive national strategy on HIV/AIDS.^126^ The resulting document, *National HIV/AIDS Strategy for the United States* (2010), set specific targets for four key objectives: reducing new HIV infections; increasing access to care and improving health outcomes for people with HIV; reducing HIV-related health disparities; and achieving a more coordinated national response to the epidemic. An *Implementation Plan* was released alongside the *Strategy*, and each relevant federal agency devised an operational plan to facilitate implementation.^127^ The *Strategy* was received with enthusiasm across multiple sectors of HIV/AIDS work.^128^ It also brought considerable media attention to the domestic epidemic.^129^ However, it was not accompanied by new budget allocations, limiting implementation to actions that could be taken by reassigning existing funds.^130^ One such objective, reflected in the fourth *Strategy* goal, involves streamlining the cumbersome and overlapping federal requirements with which community agencies and service providers must contend. ONAP itself spearheaded efforts to increase coordination, for instance, through the introduction of common requirements for grantee reporting.^131^

The 2010 *National HIV/AIDS Strategy* also shone a spotlight once more on treatment access. The 2009 CARE Act reauthorisation helped preserve treatment services for those without quality health insurance. However, the policy change likely to have the most impact on treatment access is not an HIV-specific law, but the 2010 *Patient Protection and Affordable Care Act* (ACA). Analysts estimate that up to 70 per cent of people with HIV who were uninsured before the ACA now qualify for Medicaid, and many of the remaining 30 per cent are gaining access to private insurance.^132^ This has profoundly increased the consistency of access to health care and medications. In 2008, nearly 150,000 Americans (or 10 per cent of HIV-positive Americans) were reliant on the troubled ADAPs.^133^ Under the ACA this population is rapidly moving out of a chronically underfunded, HIV/AIDS-specific programme, into more reliable, regular structures of health care provision.^134^

In addition to stabilising HIV care for many patients, HIV/AIDS services organisations assert the ACA may have two other effects. First, the law could help decrease the stigma associated with HIV by allowing most HIV-positive people to access medical treatment through traditional health care structures; and, indeed, HIV-related stigma may be ebbing, as evidenced by the recent re-examination of issues ranging from blood donation by gay men to treatment of HIV-positive inmates in prison.^135^ Second, the ACA could eventually render the CARE Act and other discretionary HIV/AIDS programmes obsolete.^136^ Although this would be a strong win in terms of reliable treatment access, it is unclear who would then pay for the social support and case management functions that CARE Act dollars currently finance.^137^

In 2015 the *Strategy* was revised to reflect substantial advancements in AIDS science and to note progress on goals made to date. The 2015 report also offered updated goals for 2020, and annual reports continue to assess the nation’s progress toward *Strategy* goals. While the long-term legacy of the *Strategy* cannot yet be determined, it is clear that the Obama years witnessed decreasing infection rates.^138^ It also paid increased attention to the HIV-related needs of highly affected US populations that previously had been neglected.^139^ During the Obama years, there was growing recognition of the role stable housing can play in preventing new infections and improving the lives of people living with HIV.^140^ In addition, there were improved efforts to ensure HIV-positive individuals are diagnosed and linked to treatment, so that they can achieve viral suppression and overall health.^141^

## Conclusion

HIV/AIDS policy can be read as a series of responses to the concrete challenges of a health crisis: preventing the virus’s spread, ensuring equitable access to treatment and facilitating research into aetiologies, treatments and cures. But HIV/AIDS policy is also a malleable political product—a resource used to wage broader social and ideological battles, as well as an instantiation of those battles. As a result, rather than evolving into a comprehensive policy that integrates the concerns of various American publics, federal HIV/AIDS policy has pursued a zig-zagging course, based on the issues that resonate best with the current party in power. Under Democratic administrations, central concerns of the political Left come to the fore: providing for the disadvantaged, expanding the health care social safety net and combatting the discrimination and stigmatisation of marginalised groups. Under Republican administrations, the epidemic is viewed through central concerns of the political Right: trimming government-funded social programmes, emphasising personal responsibility and protecting citizens and society from the negative impacts of behaviours coded as immoral.

Public health experts often view Democratic presidential administrations and Congresses as easier contexts in which to advance their preferred policy choices, particularly since Conservative leaders are more likely to accord the same weight to ideological discourses about sexuality and drug use as to scientific consensus in their policy making. So, for instance, most prevention experts strongly advocate the comprehensive approaches to sex education that research finds most effective, and such programmes generally thrived during the Clinton and Obama administrations. Under Republican administrations, prevention efforts have focused on abstinence, and grant guidelines have had a chilling effect on programmes promoting more frank and holistic approaches. Similarly, socially-conservative legislators have been steadfast in their opposition to needle exchange, which they see as conveying permission to engage in immoral behaviour. Thus, despite strong scientific consensus that needle exchange programmes reduce HIV transmission without increasing drug use, a ban on federal funding for needle exchange was enacted during the Reagan years and affirmed during the conflict-ridden Clinton administration.

This is not to say that members of the political Left never elevate their own concerns over the science-based recommendations of public health experts. Notably, in the early 2000s, the CDC determined that routine testing would benefit both those with HIV (by setting them on a road to effective treatment) and those without HIV (by clarifying the HIV status of one’s partner(s)). Nevertheless, HIV/AIDS activists resisted the transition to routine testing, out of fears that marginalised groups would face coercive testing and that individuals who tested positive would face (further) stigma and discrimination. Leaders on the political Right were less concerned about stigmatisation and more concerned with insulating the broader American public from a disease affecting primarily marginalised groups. Thus, routine testing was a relatively easy policy to implement during the Republican-led George W. Bush years.

In this fourth decade of the HIV/AIDS epidemic, state-of-the-art medical treatment has converted HIV from a short-term death sentence to a manageable, chronic disease—at least for those Americans with access to diagnosis and treatment. With no cure yet in hand, however, significant challenges remain, including, first and foremost, in the arena of treatment financing. The Affordable Care Act positions mainstream health insurance to provide comprehensive care to people with HIV for the first time, and could—if fully implemented over the long-term—bring the decades-long pursuit of HIV/AIDS-specific financing mechanisms to an end. However, the election of Republican President Donald Trump and a Republican-dominated Congress in 2016 complicates that forecast, raising the real possibility that the ACA will be dismantled, de-funded or radically revised.^142^ Even if all the provisions of the ACA were retained and implemented, challenging treatment finance questions would remain: how much should pharmaceutical companies be allowed to charge for AIDS therapies? Will mainstream health insurance markets be able to absorb the high cost of HIV/AIDS treatment? How can both medical resources and financial burdens be distributed equitably?

Beyond the treatment arena, we should expect that prevention programmes will remain particularly susceptible to political attack, since they necessarily involve politicised choices about which populations to target and how to communicate on sensitive topics. Compared to treatment and prevention, HIV/AIDS research is so far maintaining momentum: HIV/AIDS has become a major area of publicly-funded research, driven by well-established institutions that set strategic priorities and push the field aggressively forward. However, recent congressional audits illustrate the specific vulnerability of social and behavioural research on aspects of HIV/AIDS critical to stemming transmission rates and ensuring quality care for infected individuals.

Heading into the future, then, US HIV/AIDS policy advocates still have a full agenda: to continue identifying emergent problems and bringing them to policy makers’ attention; to be prepared for intense debate concerning policies that aid marginalised groups, expand government programmes, or tread the dangerous moral ground of sex and drugs; and to find the political allies and policy making windows that make it possible to advance public health.

## Funding

This work was supported by a National Science Foundation Dissertation Improvement Grant, a Wellcome Trust Travel Grant and the Reed Foundation’s Ruth Landes Memorial Research Fund.

## Footnotes

1
*New York Times (NYT)* data included all articles containing joint references to ‘AIDS’ and ‘policy’ or ‘HIV’ and ‘policy.’ *NYT* was chosen as a data source because it is one of the most widely read daily news sources in the USA, and because its entire contents are archived and searchable on LexisNexis for every day of the study period. All articles on domestic policy in the *Kaiser Daily HIV/AIDS Report* were also included. This resource was produced by the Kaiser Family Foundation, and terminated in September 2009. Many past issues of the report can be found by searching for http://www.kaisernetwork.org/daily_reports/rep_hiv.cfm at https://web.archive.org/. For the purposes of continuous data collection I replaced the Kaiser Daily HIV/AIDS report in September 2009, with the *Kaiser Health News Daily Health Policy Report* (http://www.kaiserhealthnews.org/Headlines.aspx) and the daily *POZ News Top Stories* (http://www.poz.com/archive/currentNews_1.shtml). Kaiser sources were chosen for their daily focus on HIV-related content culled from a range of news sources around the country and reported by Kaiser’s own analysts. Kaiser Family Foundation is committed to, and a respected source of, non-partisan health-news. *POZ* stories were used to supplement the Kaiser source starting from the point when Kaiser stopped producing a daily report focused solely on HIV. News data were analysed by summarising the main content of each individual news story; cataloguing and sorting stories by year and theme using extensive Excel spreadsheets; and analysing patterns across themes and across time.

2 Legislative histories were gathered from the *Congressional Universe* database.

3 Interviews were conducted specifically for this historical project, and for the accompanying book-length project explaining similarities and differences between HIV/AIDS policy decisions in the USA and the UK. Interviews averaged about 90 minutes; most were conducted in person, the remainder by phone. Interviews followed a semi-structured format, in which informants reflected in their own words on their policy making work and the larger contexts of national policy debates and decisions. Most informants spoke confidentially and are thus cited using unique identification numbers; others waived confidentiality and chose instead to be identified by name. Interview data were analysed using the inductive methods of thematic coding and constant comparison, with the aid of NVivo software for qualitative data. Barney G. Glaser and Anselm L. Strauss, eds, *The Discovery of Grounded Theory: Strategies for Qualitative Research* (Chicago, IL: Aldine Publishing Company, 1967).

4 These include agency reports, hearing and debate transcripts, congressional and parliamentary testimony, executive orders, issue briefs, advocacy guides and meeting notes. Many of the documents are publicly available; the remainder were provided by study informants.

5 This article issues from a parent project that pursued parallel data collection and analysis in the USA and UK, in order to contextualise US HIV/AIDS policy choices within a broader range of options.

6 Centers for Disease Control and Prevention, ‘HIV in the United States: At a Glance’, *HIV/AIDS*, 25 November 2014, <http://www.cdc.gov/hiv/statistics/basics/ataglance.html>.

7 On NIH spending, see Jack Whitescarver, ‘Trans-NIH AIDS Research By-Pass Budget Estimate and Trans-NIH Plan for HIV-Related Research FY 2013’ (Office of AIDS Research, National Institutes of Health, 2013), <https://www.oar.nih.gov/sites/default/files/FY2013ByPassPlan_508_Full.pdf>.

8 In late 1982 US and international communities settled on the name Acquired Immune Deficiency Syndrome (AIDS) for the new disease. Before that, it was known by other names, most prominently Gay-Related Immune Disease (GRID) and—in the press—the ‘gay plague’. The term HIV came into use after the viral origin of the disease was discovered. HIV/AIDS is now the most commonly used term, with AIDS used to refer specifically to the symptomatic end-stage of the disease and HIV-disease used to refer to the larger course of disease beginning at the time of HIV infection.

9 Mirko D. Grmek, *History of AIDS: Emergence and Origin of a Modern Pandemic* (Princeton, NJ: Princeton University Press, 1990); Jacques Pepin, *The Origins of AIDS* (Cambridge: Cambridge University Press, 2011). Other sources argue for slightly different benchmark dates for first recognition of the problem.

10 HIV.gov, ‘A Timeline of HIV and AIDS’, n.d., https://www.hiv.gov/hiv-basics/overview/history/hiv-and-aids-timeline>; Edward King, ed., *Safety in Numbers: Safer Sex and Gay Men* (New York: Routledge, 1993).

11 The association of HIV/AIDS with marginalised and stigmatised social groups continued to complicate and impede policy responses during the later history of the epidemic as well; disproportionate numbers of infections among African Americans and injecting drug users have repeatedly facilitated ‘victim-blaming’ narratives associated with the de-prioritisation of HIV/AIDS issues.

12 Sandra Panem, ed., *The AIDS Bureaucracy* (Cambridge, MA: Harvard University Press, 1988). The high level of mortality was due to a standard two-year waiting period between diagnosis of a recognised disability and eligibility for benefits under Medicaid.

13 King, *Safety in Numbers: Safer Sex and Gay Men*.

14 See Julia A. Ericksen and Sally A. Steffen, *Kiss and Tell: Surveying Sex in the Twentieth Century* (Cambridge, MA: Harvard University Press, 2001); Randy Shilts, ed., *And the Band Played on: Politics, People, and the AIDS Epidemic* (New York: Penguin Books, 1987).

15 For a rich accounting of this period, see Dennis Altman, ‘Legitimation Through Disaster: AIDS and the Gay Movement’, in Elizabeth Fee and D. M. Fox, eds, *AIDS: The Burdens of History* (Berkeley, CA: University of California Press, 1988), 301–15; Dennis Altman, ‘AIDS: The Burdens of History’,’ in Elizabeth Fee and Daniel M. Fox, eds, *Legitimation through Disaster: AIDS and the Gay Movement* (Berkeley, CA: University of California Press, 1988), 301–15; Ronald Bayer and David L. Kirp, ‘Introduction: An Epidemic in Political and Policy Perspective’,’ in Kirp and Bayer, eds, *AIDS in the Industrialized Democracies: Passions, Politics, and Policies* (New Brunswick, NJ: Rutgers University Press, 1992), 1–6; Ronald Bayer and David L. Kirp, ‘The United States: At the Center of the Storm’, in Kirp and Bayer, eds, *AIDS in the Industrialized Democracies* 7–48; Allan M. Brandt, ‘AIDS: From Social History to Social Policy’, *Law, Medicine & Health Care*, 1986, 14, 231–42; Jennifer Brier, *Infectious Ideas: U.S. Political Responses to the AIDS Crisis* (Chapel Hill, NC: University of North Carolina Press, 2009); Neal Arthur Dickerson, ed., *The Promise and the Power: Public Policy Analysis and the AIDS Movement* (Las Colinas, CA: Monument Press, 1993); Steven Epstein, ed., *Impure Science : AIDS, Activism, and the Politics of Knowledge* (Berkeley, CA: University of California Press, 1996); Daniel M. Fox, ‘The Politics of HIV Infection: 1989–1990 as Years of Change’, in Elizabeth Fee and Daniel M. Fox, eds, *AIDS: The Making of a Chronic Disease* (Berkeley, CA: University of California Press, 1992), 125–43; Panem, *The AIDS Bureaucracy*; Michael Quam and Nancy Ford, ‘AIDS Policies and Practices in the United States’, in Barbara A. Misztal and David Moss, eds, *Action on AIDS: National Policies in Comparative Perspective* (Westport, CT: Greenwood Press, 1990), 25–50; Shilts, *And the Band Played On*.

16 For the rising levels of demand on health services, see Victoria A. Harden, *AIDS at 30: A History* (Washington, DC: Potomac Books, 2012); C. Everett Koop, ‘The Early Days of AIDS, as I Remember Them’, *Annals of the Forum for Collaborative HIV Research*, 2011, 13, 5–10.

17 Institute of Medicine and National Academy of Sciences, *Confronting AIDS: Directions for Public Health, Health Care, and Research* (Washington, DC: National Academy Press, October 1986); C. Everett Koop, ‘Surgeon General’s Report on Acquired Immune Deficiency Syndrome’ (Washington, DC: U.S. Public Health Service Public Affairs Office, October 1986); Koop, ‘The Early Days of AIDS, as I Remember Them’; Robin Weiss and Samuel O. Thier, ‘The Institute of Medicine, National Academy of Sciences: Formulating AIDS Policy’, *Public Health Reports*, 1988, 103, 289–92. In its summaries of the crisis cities were facing, the IOM/NAS echoed strong themes that had been raised by urban hospital leaders during Congressional hearings earlier in the decade. See for example: House of Representatives Human Resources and Intergovernmental Relations Subcommittee of the Committee on Government Operations, *The AIDS Epidemic in Newark and Detroit* (Washington, DC: Government Printing Office, 1989).

18 Virginia Berridge, ed., *AIDS in the UK: The Making of Policy, 1981–1994* (New York: Oxford University Press, 1996).

19 Tasleem J. Padamsee, ‘Infusing Health into the Welfare State: AIDS Policy Making in the United States and the United Kingdom’ (Michigan: University of Michigan, 2007).

20 A globally coordinated response began in 1987, with the launch of the World Health Organization (WHO) Global Programme on AIDS. Subsequently, UNAIDS and other supra-national organisations became major players in HIV/AIDS policy making around the world. The USA has participated actively in the global response; however, global HIV/AIDS policy has not exerted reciprocal influence on US domestic policy and therefore is not discussed here. For an informative review, see UNAIDS, ‘Report on the Global AIDS Epidemic’, 2008, <http://www.unaids.org/sites/default/files/media_asset/jc1510_2008globalreport_en_0.pdf>.

21 Barbara A. Misztal and David Moss, ‘Conclusion’, in Misztal and Moss, eds., *Action on AIDS: National Policies in Comparative Perspective*, (Westport, CT: Greenwood Press, 1990), 235–50; Quam and Ford, ‘AIDS Policies and Practices in the United States’.

22 Harden, *AIDS at 30: A History*; Koop, ‘The Early Days of AIDS, as I Remember Them.’

23 Claire Elizabeth Ernst, ‘Taking Exception: AIDS, Civil Rights, and Public Health in France and the United States, 1981–1990’ (New York: Cornell University, 1994); Bayer and Kirp, ‘The United States: At the Center of the Storm’.

24 Quam and Ford, ‘AIDS Policies and Practices in the United States’.

25 Senator Jesse (NC) Helms, ‘Amendment 963,’ *Congressional Record*, 1987, 133, 27766.

26 Officially, the provisions of the Helms Amendment applied only to federally-funded materials and activities. However, its effects were more widespread, as the CDC (which funded most of the large AIDS organisations) enacted broad guidelines to protect its programmes, and CBOs feared that even privately-funded materials/activities would attract harmful federal scrutiny. Nor were their fears misplaced, given that Helms’ original critique of ‘homoerotic literature’ in HIV/AIDS prevention work was based on materials produced exclusively with private funds. Peter Lewis Allen, ed., *The Wages of Sin: Sex And Disease, Past and Present* (Chicago, IL: University of Chicago, 2000), excerpt, ‘The Birth of the Helms Amendment: How a Single Pamphlet Started an AIDS War’ at: <https://supreme.findlaw.com/legal-commentary/the-birth-of-the-helms-amendment.html>; Ernst, ‘Taking Exception: AIDS, Civil Rights, and Public Health in France and the United States, 1981–1990’; King, *Safety in Numbers: Safer Sex and Gay Men*; Quam and Ford, ‘AIDS Policies and Practices in the United States’; Aran Ron and David E. Rogers, ‘AIDS in the United States: Patient Care and Politics’, *Daedalus*, 1989, 118, 41–58.

27 Tasleem J. Padamsee, ‘The Politics of Prevention: Lessons from the Neglected History of U.S. HIV/AIDS Policy’, *Journal of Health Politics, Policy, and Law*, 2017, 42, 73–122; Sean Cahill and Nathan Schaefer, eds, ‘Syringe Exchange Programs around the World: The Global Context’ (Gay Men's Health Crisis, 2009), <http://www.gmhc.org/files/editor/file/gmhc_intl_seps.pdf>.

28 In both cases, funding for needle exchange programmes could have been filtered through state or regional public health departments.

29 Padamsee, ‘The Politics of Prevention: Lessons from the Neglected History of U.S. HIV/AIDS Policy’, 2017.

30 Virginia Berridge, ‘AIDS and British Drug Policy: A Post-War Situation?’, in Michael Bloor and Fiona Wood, eds, *Addictions and Problem Drug Use: Issues in Behaviour, Policy, and Practice*, Research Highlights in Social Work 33 (London: Jessica Kingsley Publishers, 1998), 85–106.

31 Berridge, *AIDS in the UK: The Making of Policy, 1981–1994*; John Street and Albert Weale, ‘AIDS in the Industrialized Democracies: Passions, Politics, and Policies’, in David L. Kirp and Ronald Bayer, eds, *Britain: Policy-Making in a Hermetically Sealed System* (New Brunswick, NJ: Rutgers University Press, 1992), 185–220.

32 Berridge, ‘AIDS and British Drug Policy: A Post-War Situation?’; Gerry V. Stimson, ‘AIDS and Drug Misuse: The Challenge for Policy and Practice in the 1990s’, in John Strang and Gerry V. Stimson, eds, *Revising Policy and Practice: New Ideas about the Drugs Problem* (London and New York: Routledge, 1990), 121–31; Street and Weale, ‘Britain: Policy-Making in a Hermetically Sealed System’; Aviva Bresky, Interview with Author, 2005; Hilary Curtis, Interview with Author, 2005.

33 For discussion of the role of scientific expertise in American versus European policy making more generally, see Sheila Jasanoff, ed., *The Fifth Branch: Science Advisers as Policymakers* (Cambridge, MA: Harvard University Press, 1998).

34 Victor F. Zonana, ‘Sullivan Shoots Down Plans to Supply Needles’, *Los Angeles Times*, 20 July 1989, <http://articles.latimes.com/1989-07-20/news/mn-5182_1_needle-exchange>.

35 Bradley M. Mathers *et al.*, ‘Global Epidemiology of Injecting Drug Use and HIV among Peole Who Inject Drugs: A Systematic Review’, *The Lancet*, 2008, 372, 1733–45.

36 ACT-UP’s Treatment Action Committee later became the independent Treatment Action Group.

37 Brier, *Infectious Ideas: U.S. Political Responses to the AIDS Crisis*; ‘1993 Revised Classification System for HIV Infection and Expanded Surveillance Case Definition for AIDS among Adolescents and Adults’, *MMWR. Recommendations and Reports: Morbidity and Mortality Weekly Report. Recommendations and Reports* 41, no. RR-17 (18 December 1992), 1–19; Epstein, *Impure Science : AIDS, Activism, and the Politics of Knowledge*.

38 Bayer and Kirp, ‘The United States: At the Center of the Storm’; Ernst, ‘Taking Exception: AIDS, Civil Rights, and Public Health in France and the United States, 1981–1990’.

39 Panem, *The AIDS Bureaucracy*.

40 National Commission on Acquired Immune Deficiency Syndrome, ‘America Living with AIDS: Transforming Anger, Fear, and Indifference into Action’, in Philip R. Lee and Carroll L. Estes, eds, *The Nation’s Health*, 4th edn (Boston, MA: Jones and Barlett, 1994), 391–7; ‘AIDS Panel Head Calls U.S. Efforts Inadequate’, *New York Times*, 2 November 1992, sec. B10.

41 Robin Weiss and Leslie M. Hardy, ‘HIV Infection and Health Policy’, *Journal of Consulting and Clinical Psychology*, 1990, 58, 70–6.

42 Shilts, *And the Band Played on: Politics, People, and the AIDS Epidemic*.

43 Although it is widely known that a significant proportion of the HIV/AIDS population was under- or uninsured, data for the late 1980s and early 1990s do not exist. In 1996, 20% had no insurance, private or public; in 2002, this figure was 28%. Samuel A. Bozzette *et al.*, ‘The Care of HIV-Infected Adults in the U.S.’, *New England Journal of Medicine*, 1998, 339, 1897–904; John A. Fleishman *et al.*, ‘Hospital and Outpatient Health Services Utilization among HIV-Infected Adults in Care 2000–2002’, *Medical Care*, 2005, 43, III-40–III-52; Jennifer Kates, ‘Personal Communication’, 2007; Jennifer Kates and Jeffrey Levi, ‘Where Do Americans Access Care? Considerations for Testing and Planning for Additional HIV-Infected People in the Health Care System’ (Opportunities for Improving HIV Diagnosis, Prevention, and Access to Care in the U.S., Conference Presentation, Washington, DC, 2006).

44 Confidential Informant #5, Interview with Author, 2005.

45 House of Representatives Human Resources and Intergovernmental Relations Subcommittee of the Committee on Government Operations, *The AIDS Epidemic in Newark and Detroit* (Washington, DC: Government Printing Office, 1989).

46 Jeffrey Levi, Interview with Author, 2005; Julie Scofield, Interview with Author, 2005; Confidential Informant #5, Interview with Author, 2005; Confidential Informant #6, Interview with Author, 2005.

47 The aerosolised pentamidine treatments that emerged in the late 1980s helped stave off deadly pneumonias in AIDS patients, and research seemed to show that AZT might delay the onset of full-blown AIDS and death. Confidential Informant #6, Interview with Author, 2005.

48 Tasleem J. Padamsee, ‘Infusing Health into the Welfare State: AIDS Policy Making in the United States and the United Kingdom’ (Michigan: University of Michigan, 2007).

49 In 1987 the UK instituted HIV/AIDS-specific treatment allocations for every National Health Service region.

50 Confidential Informant #6, Interview with Author, 2005.

51 The formulation of a method to cover *all* members of a particular group was particularly striking in the pre-ACA era. It is useful to recall that, in 1990, over 16% of the US population was uninsured, and insurance companies were legally permitted to deny coverage to individuals with pre-existing conditions. (For further examination of the ACA and AIDS, see discussion of the Obama presidency below.) Robin A. Cohen *et al.*, *Health Insurance Coverage Trends, 1959–2007: Estimates from the National Health Interview Survey* (Department of Health and Human Services, Centers for Disease Control and Prevention, National Center for Health Statistics, 2009), <https://pdfs.semanticscholar.org/9a83/b80c9247ddfb51082360ec8759f379e43625.pdf>.

52 ADA protections with respect to HIV were significantly strengthened in subsequent years; the ADA now protects persons at all stages of HIV disease, as well as people suspected of being infected or at risk of being infected, from discrimination. ‘AIDS: Covered Indirectly, Americans with Disabilities Act’, *Congressional Quarterly Weekly Report* 47 (13 May 1989), 1123; Derek Adam-Smith and Fiona Goss, ‘AIDS: Activism and Alliances’, in Peter Aggleton, Graham Hart and Peter Davies, eds, *Opportunity Lost: HIV/AIDS, Disability and Legislation* (London, UK: Taylor & Francis, 1997), 25–40; Bayer and Kirp, ‘The United States: At the Center of the Storm’; Gerald J. Stine, ‘Acquired Immune Deficiency Syndrome: Biological, Medical, Social, and Legal Issues’, in Gerald J. Stine, eds., *Legal Aspects of HIV/AIDS: A Review of Legislation and Court Decisions in the United States* (Englewood Cliffs, NJ: Prentice Hall, 1993), 393–430.

53 HOPWA services include short- and long-term rental assistance and grants to develop and operate housing for people with AIDS Department of Housing and Urban Development, ‘Housing Opportunities for People with AIDS (HOPWA) Program’, 2007, <https://www.hudexchange.info/programs/hopwa/>.

54 David Holtgrave, Interview with Author, 2011.

55 National Commission on Acquired Immune Deficiency Syndrome, ‘AIDS, An Expanding Tragedy’ (Washington, D.C.: National Commission on Acquired Immune Deficiency Syndrome, 1993); Levi, Interview with Author, 2005; Sandra Thurman, Interview with Author, 2011; Confidential Informant #6, Interview with Author, 2005; Confidential Informant #2, Interview with Author, 2005.

56 Kristine Gebbie, Interview with Author, 2005; Confidential Informant #5, Interview with Author, 2005; Confidential Informant #2, Interview with Author.

57 Gebbie, Interview with Author; Brent Minor, Interview with Author, 2005; Confidential Informant #5, Interview with Author, 2005; Confidential Informant #27, Interview with Author, 2005.

58 Presidential Advisory Council on HIV/AIDS, ‘Second Progress Report, December 7’ (Washington, DC: U.S. Centers for Disease Control and Prevention, 1997), <http://www.thebody.com/cdc/prezsum.html#preamble>; Presidential Advisory Council on HIV/AIDS, ‘AIDS—No Time to Spare: The Final Report to the President of the United States’ (Washington, DC: Centers for Disease Control and Prevention National Prevention Information Network, September 2000).

59 Todd Summers, Priya Alagiri and Jennifer Kates, ‘Federal HIV/AIDS Spending: A Budget Chartbook, Fiscal Year 2002,’ September 2003, <http://kaiserfamilyfoundation.files.wordpress.com/2013/01/federal-hiv-aids-spending-a-budget-chartbook-fiscal-year-2002-pdf.pdf>.

60 John Henkel, ‘Attacking AIDS with a “Cocktail” Therapy: Drug Combo Sends Deaths Plummeting’, *FDA Consumer Magazine*, August 1999, <https://aidsinfo.nih.gov/news/493/attacking-aids-with-a-cocktail-therapy--drug-combo-sends-deaths-plummeting>; Minor, Interview with Author. The annual US death toll from AIDS rose steadily from 1981 to 1995, peaking at 51,414. In 1996, due to HAART, the death count fell to 38,074 and continued to decline thereafter, even as the HIV-positive population grew. In 2005, 17,011 Americans died from AIDS. Centers for Disease Control and Prevention, ‘U.S. HIV and AIDS Cases Reported through December 2001’, HIV/AIDS Surveillance Report, Year-End Edition (Washington, DC: Centers for Disease Control and Prevention, 2001), <http://www.cdc.gov/hiv/pdf/statistics_2001_HIV_Surveillance_Report_vol_13_no2.pdf>; Centers for Disease Control and Prevention, ‘Cases of HIV Infection and AIDS in the United States and Dependent Areas, 2007’, HIV Surveillance Report, 2007, <http://www.cdc.gov/hiv/pdf/statistics_2007_HIV_Surveillance_Report_vol_19.pdf>.

61 Peter S. Arno et al., ‘Economic and Policy Implications of Early Intervention in HIV Disease’, *Journal of the American Medical Association*, 1989, 262, 1493–98; Brian Haile, ‘Developments in Policy and Health Services Research in HIV/AIDS’, *Journal of Compensation & Benefits*, 2001, 17, 40–2; J. G. Kahn *et al.*, ‘Health and Federal Budgetary Effects of Increasing Access to Antiretroviral Medications for HIV by Expanding Medicaid’, *American Journal of Public Health*, 2001, 91, 1464–73; Stephen F. Morin and Edwin D. Charlebois, ‘Expanding Access to Early HIV Care: New Challenges for Federal Health Policy’, *AIDS & Public Policy Journal*, 15, 65–74.

62 Kaiser Family Foundation, ‘Kaiser Daily HIV/AIDS Report’, 1995, 1996.

63 Institute of Medicine Committee on Perinatal Transmission of HIV, *Reducing the Odds: Preventing Perinatal Transmission of HIV in the United States* (Washington, DC: National Academy Press, 1998).

64 Kaiser Family Foundation, ‘Kaiser Daily HIV/AIDS Report’, 2000.

65 ‘Ryan White & AIDS Drug Assistance Programs’, *National Alliance of State & Territorial AIDS Directors*, <https://www.nastad.org/domestic/health-care-access/ryan-white-aids-drug-assistance-programs>, accessed 8 December 2016; Kaiser Family Foundation, ‘Kaiser Daily HIV/AIDS Report’, 1996, 1997, 1998, 2000, and 2001. NASTAD: National Association of State and Territorial AIDS Directors, ‘ADAP Watch Reports (Monthly & Annual)’, *Ryan White & AIDS Drug Assistance Programs*, 2016, <https://www.nastad.org/domestic/health-care-access/ryan-white-aids-drug-assistance-programs>.

66 Minor, Interview with Author; Confidential Informant #13, Interview with Author, 2005; Confidential Informant #19, Interview with Author, 2011; NASTAD: National Association of State and Territorial AIDS Directors, ‘ADAP Watch Reports (Monthly & Annual)’.

67 Kaiser Family Foundation, ‘Kaiser Daily HIV/AIDS Report’, 1995, 1998, and 1999.

68 For the 1993 disability rules, see Department of Health and Human Services, ‘Federal Old-Age, Survivors, and Disability Insurance; Determining Disability and Blindness; Revision of Part A and Part B of the Listing of Impairments, Endocrine, and Multiple Body Systems; Immune System (Final Rules; 58 FR 36008, July 2, 1993)’, <https://www.ssa.gov/OP_Home/hallex/II-04/II-4-1-4.html>. On the 1999 Ticket to Work Incentives Improvement Act, see Haile, ‘Developments in Policy and Health Services Research in HIV/AIDS’; ‘Public Law 106–170—Ticket to Work and Work Incentives Improvement Act of 1999’, 1999, <https://www.gpo.gov/fdsys/pkg/PLAW-106publ170/content-detail.html>.

69 Confidential Informant #6, Interview with Author, 2005; Kaiser Family Foundation, ‘Kaiser Daily HIV/AIDS Report’, 1998, 2000, 2001, 2003, and 2004.

70 Minor, Interview with Author; Confidential Informant #13, Interview with Author, 2005; Kaiser Family Foundation, ‘Kaiser Daily HIV/AIDS Report’, 1997 and 1998. Confidential Informant #11, Interview with Author, 2005; Confidential Informant #30, Interview with Author, 2005. In the absence of a comprehensive, federal solution, advocates pursued a piecemeal strategy, seeking federal waivers to allow states to cover people with HIV in their Medicaid programmes. Some scholars argued that this strategy would ultimately prove more productive than efforts at federal reform. However, waivers were only granted where states could prove their expanded programmes would be federally cost-neutral, and, prior to the ACA (see below), only Mississippi, Maine, Massachusetts and Washington, DC had active expansion programmes. Kaiser Family Foundation, ‘Status of State Medicaid Expansion Efforts for People with HIV, May 2004’, 2004, <http://www.statehealthfacts.org/cgi-bin/healthfacts.cgi?action=compare&category=HIV%2FAIDS&subcategory=Other+HIV%2FAIDS-Related+Policies&topic=State+Medicaid+Expansion+Efforts> (accessed on 15 December, 2015); Haile, ‘Developments in Policy and Health Services Research in HIV/AIDS’.

71 Judith Auerbach, Interview with Author, 2005; Neal Nathanson, Interview with Author, 2005; Wendy Wertheimer, Interview with Author, 2011; Jack Whitescarver, Interview with Author, 2011; Confidential Informant #20, Interview with Author, 2011.

72 In 1995, the House of Representatives tried unsuccessfully to strip the OAR’s power; in 1996, the blue-ribbon Levine Committee advocated for stronger OAR control. Despite variations over time, the OAR remains a guiding force in AIDS research. Nathanson, Interview with Author; Confidential Informant #20, Interview with Author, 2005; Confidential Informant #32, Interview with Author, 2005.

73 Alison Mitchell, ‘Clinton Calls for AIDS Vaccine as Goal’, *The New York Times*, 19 May 1997, sec. B8; Kaiser Family Foundation, ‘Kaiser Daily HIV/AIDS Report’, 1997, 1998, and 2000.

74 Jean McGuire, ‘The Emergence of AIDS: The Impact on Immunology, Microbiology and Public Health’, in Kenneth H. Mayer and H. F. Pizer, eds, *Inclusion, Representation, and Parity: The Making of a Public Health Response to HIV* (Washington, DC: American Public Health Association, 2000), 181–205; Confidential Informant #2, Interview with Author; Confidential Informant #33, Interview with Author, 2005; Confidential Informant #162, Interview with Author, 2011.

75 On injecting drug use as a causal factor, see Presidential Advisory Council on HIV/AIDS, ‘Meeting Minutes, December’, 1995. On criteria for lifting the ban, see Warwick Anderson, ‘The New York Needle Trial: The Politics of Public Health in the Age of AIDS’, *American Journal of Public Health*, 1991, 81, 1506–17; Jacques Normand, David Vlahov and Lincoln E. Moses, ‘Preventing HIV Transmission: The Role of Sterile Needles and Bleach’, National Research Council and Institute of Medicine (Washington, DC: National Academies Press, 1995); Presidential Advisory Council on HIV/AIDS, ‘Meeting Minutes, July’, 1995; Presidential Advisory Council on HIV/AIDS, ‘Meeting Minutes, September’, 1995; Presidential Advisory Council on HIV/AIDS, ‘Meeting Minutes, December’, 1996.

76 Gebbie, Interview with Author; Confidential Informant #5, Interview with Author, 2005; Confidential Informant #22, Interview with Author, 2005; Confidential Informant #27, Interview with Author; Confidential Informant #29, Interview with Author, 2005; Confidential Informant #33, Interview with Author; Presidential Advisory Council on HIV/AIDS, ‘Meeting Minutes, December 1995—March 1998’, December 1995; Presidential Advisory Council on HIV/AIDS, ‘Letter to Secretary of Health & Human Services Donna Shalala, December 17’, 1996; Presidential Advisory Council on HIV/AIDS, ‘First Progress Report, July 8’, 1996; Presidential Advisory Council on HIV/AIDS, ‘Letter to President William Jefferson Clinton, December 7’, 1997; Presidential Advisory Council on HIV/AIDS, ‘Meeting Minutes, December’, 1995; Presidential Advisory Council on HIV/AIDS, ‘Letter to Secretary of Health & Human Services Donna Shalala, March 16’, 1998; Presidential Advisory Council on HIV/AIDS, ‘Letter to President William Jefferson Clinton, March 17’, 1998.

77 The odd formulation was driven in part by congressional Democrats, who feared for their political futures, should the ban later be rescinded. Padamsee, ‘The Politics of Prevention: Lessons from the Neglected History of U.S. HIV/AIDS Policy’, 2017; Gebbie, Interview with Author; Gregg Gonsalves, Interview with Author, 2005; Levi, Interview with Author, 2005; Confidential Informant #5, Interview with Author, 2005; Confidential Informant #6, Interview with Author, 2005; Confidential Informant #8, Interview with Author, 2005; Confidential Informant #10, Interview with Author, 2005; Confidential Informant #29, Interview with Author, 2005; Health and Human Services Press Office, ‘Research Shows Needle Exchange Programs Reduce HIV Infections Without Increasing Drug Use’ (Press Release, 20 April 1998), <https://www.csam-asam.org/needle-exchange-programs-reduce-hiv-infections>; Presidential Advisory Council on HIV/AIDS, ‘Meeting Minutes, December’, 1997.

78 Because Congress must approve the District of Columbia’s annual budget, the District’s desire to implement needle exchange programmes provided the most prominent ongoing venue for this debate at the federal level. Kaiser Family Foundation, ‘Kaiser Daily HIV/AIDS Report’, 2000, 2001, 2002; As of 2002, 31 states and Washington, DC officially allowed needle exchange programmes. ‘Sterile Syringe Exchange Programs, 2002’, n.d., <http://www.statehealthfacts.org/cgi-bin/healthfacts.cgi?previewid=252&action=compare&category=HIV%2fAIDS&subcategory=Other+HIV%2fAIDS%2dRelated+Policies&topic=Syringe+Exchange+Programs&link_category=&link_subcategory=&link_topic=&viewas=&showregions=0&sortby=&datatype=&from=none&welcome=0&printerfriendly=1> (accessed on 15 December, 2015).

79 For example, over 45% of AIDS deaths in the USA had occurred among people of colour in every year between 1987 and 1990. Centers for Disease Control and Prevention, ‘U.S. AIDS Cases Reported through December 1990’, HIV/AIDS Surveillance, Year-End Edition (Washington, DC: Centers for Disease Control and Prevention, January 1991), <https://www.cdc.gov/hiv/pdf/library/reports/surveillance/cdc-hiv-surveillance-report-1990-vol-3.pdf>; Centers for Disease Control and Prevention, ‘U.S. AIDS Cases Reported through December 1991’, HIV/AIDS Surveillance, Year-End Edition (Washington, DC: Centers for Disease Control and Prevention, February 1994), <https://www.cdc.gov/hiv/pdf/library/reports/surveillance/cdc-hiv-surveillance-report-1994-vol-6-2.pdf>.

80 Warren E. Leary, ‘Commission Says Racism Contributes to AIDS Spread’, *New York Times*, 12 January 1993, sec. A14; National Commission on Acquired Immune Deficiency Syndrome, ‘AIDS, An Expanding Tragedy’.

81 The behaviour of federal health authorities up to this point reflected the general lack of awareness of the growing problems HIV/AIDS posed within minority communities. In 1991, for instance, the CDC cut funding to 23 of its 27 national AIDS prevention programmes within minority communities. AIDS Project Los Angeles, ‘Timeline of the Epidemic’, n.d., <http://www.apla.org/facts/timeline.html> (accessed on 15 December, 2010).

82 Cornelius Baker, Interview with Author, 2011; Donna Crews, Interview with Author, 2011; Confidential Informant #26, Interview with Author, 2005; Confidential Informant #129, Interview with Author, 2011.

83 Paul E. Farmer, David A. Walton and Jennifer J. Furin, ‘The Emergence of AIDS: The Impact on Immunology, Microbiology and Public Health’, in Kenneth H. Mayer and H. F. Pizer, eds, *The Changing Face of AIDS: Implications for Policy and Practice* (Washington, DC: American Public Health Association, 2000), 139–61; Confidential Informant #26, Interview with Author; Confidential Informant #27, Interview with Author.

84 See Centers for Disease Control and Prevention, ‘30 Years of HIV in African American Communities: A Timeline’, n.d., <https://www.cdc.gov/nchhstp/newsroom/docs/timeline-30years-hiv-african-american-community-508.pdf>; Confidential Informant #26, Interview with Author; Confidential Informant #27, Interview with Author; Kaiser Family Foundation, ‘Kaiser Daily HIV/AIDS Report’, 1998 and 2000. The phrase ‘epidemic of colour’ was coined by Surgeon General David Satcher in 1998. On the policy challenge specifically, see Centers for Disease Control and Prevention, ‘HIV/AIDS Prevention at CDC’, 2007, <http://www.cdc.gov/hiv/aboutDHAP.htm#strategy>; White House Office of National AIDS Policy, ‘National HIV/AIDS Strategy for the United States’ (Washington, DC: White House, 2010). For instance, in 2014, African Americans represented 12% of the US population but 44% of new HIV diagnoses and 48% of people living with AIDS. Their viral suppression rate was 21%, compared to 30% for Whites. In 2011, Hispanic Americans represented 17% of the US population; 21% of new HIV diagnoses; and 20% of those living with AIDS. Their viral suppression rate was 26%, worse than for Whites but better than for African Americans. Lawrence K. Altman, ‘U.S. Blacks, If a Nation, Would Rank High on AIDS’, *New York Times*, 30 December 2008, sec. A10; Kaiser Family Foundation, ‘Kaiser Daily HIV/AIDS Report’, 1998, 2008, 2009; Kaiser Family Foundation, ‘Black Americans and HIV/AIDS’, *Disparities Policy*, 2014; Centers for Disease Control and Prevention, ‘HIV Among African Americans’, 2016, <http://www.cdc.gov/hiv/group/racialethnic/africanamericans/index.html>; Centers for Disease Control and Prevention, ‘HIV Surveillance by Race/Ethnicity (through 2015)’, 2015, <http://www.cdc.gov/hiv/library/slidesets/index.html>.

85 Kathryn Carovano, ‘More than Mothers and Whores: Redefining the AIDS Prevention Needs of Women,’ *International Journal of Health Services* 21, no. 1 (1991): 131–42; Cindy Patton, ed., *Inventing AIDS* (New York, NY: Routledge, 1990); Paula A. Treichler, ‘How to Have Theory in an Epidemic,’ in *AIDS and HIV Infection in the Third World: A First World Chronicle*, ed. Paula A. Treichler (Durham, NC: Duke University Press, 1999), 99–126.

86 Treichler, ‘AIDS and HIV Infection in the Third World: A First World Chronicle’; Patton, *Inventing AIDS*; Carovano, ‘More than Mothers and Whores: Redefining the AIDS Prevention Needs of Women’.

87 Treichler, ‘AIDS and HIV Infection in the Third World: A First World Chronicle’; Patton, *Inventing AIDS*; Carovano, ‘More than Mothers and Whores: Redefining the AIDS Prevention Needs of Women’.

88 Padamsee, ‘The Politics of Prevention: Lessons from the Neglected History of U.S. HIV/AIDS Policy’, 2017; Carovano, ‘More than Mothers and Whores: Redefining the AIDS Prevention Needs of Women’; Patton, *Inventing AIDS*.

89 Carovano, ‘More than Mothers and Whores: Redefining the AIDS Prevention Needs of Women’; Robin Gorna, ed., *Vamps, Virgins and Victims: How Can Women Fight AIDS?* (London, UK: Cassell, 1996); Treichler, ‘AIDS and HIV Infection in the Third World: A First World Chronicle’.

90 Treichler, ‘AIDS and HIV Infection in the Third World: A First World Chronicle’.

91 Brier, *Infectious Ideas: U.S. Political Responses to the AIDS Crisis*; Carovano, ‘More than Mothers and Whores: Redefining the AIDS Prevention Needs of Women’; Gorna, *Vamps, Virgins and Victims: How Can Women Fight AIDS?*; Evelynn Hammonds, ‘Missing Persons: African-American Women, AIDS, and the History of Disease’, *Radical America*, 1992, 24, 7–23; Patton, *Inventing AIDS*; Treichler, ‘AIDS and HIV Infection in the Third World: A First World Chronicle’; Evelynn Hammonds, ‘Race, Sex, AIDS, and the Construction of Other’, *Radical America*, 1987, 20, 28–38; Leslie Wolfe, Interview with Author, 2005; Confidential Informant #2, Interview with Author.

92 Brier, *Infectious Ideas: U.S. Political Responses to the AIDS Crisis*; Neal Arthur Dickerson, ed., *Redefining AIDS: The Dead and Dispossessed, The Politics of AIDS* (Las Colinas, CA: Monument Press, 1994); Cathy Olufs, ‘The AIDS Timeline: 20 Years, 20 Drugs, 20 Million Deaths; No Cure in Sight’, 2001, <https://www.ncbi.nlm.nih.gov/pubmed/11682998>; Treichler, ‘AIDS and HIV Infection in the Third World: A First World Chronicle’.

93 For the study of HIV in women, see ‘HIV Pathogenesis in Women’s Interagency HIV Study (WIHS)’, *NIH Guide* 26, no. 30 (5 September 1997), <http://grants.nih.gov/grants/guide/pa-files/PA-97-105.html>. ‘NIH Guidelines on the Inclusion of Women and Minorities as Subjects in Clinical Research’, *NIH Guide* 23, no. 11 (18 March 1994), <http://grants.nih.gov/grants/guide/notice-files/not94-100.html>.

94 Wolfe, Interview with Author; Confidential Informant #2, Interview with Author.

95 Centers for Disease Control and Prevention, ‘HIV in the United States: At a Glance’; Centers for Disease Control and Prevention, ‘HIV/AIDS Surveillance in Women (through 2011)’, *HIV/AIDS Slide Sets*, 3 November 2014, <http://www.cdc.gov/hiv/topics/surveillance/resources/slides/women/index.htm>; Centers for Disease Control and Prevention, ‘Diagnoses of HIV Infection in the United States and Dependent Areas’, HIV Surveillance Report, Vol. 28, 2016, <https://www.cdc.gov/hiv/pdf/library/reports/surveillance/cdc-hiv-surveillance-report-2016-vol-28.pdf>.

96 Minor, Interview with Author; Rhon Reynolds, Interview with Author, 2005; Confidential Informant #10, Interview with Author; Confidential Informant #23, Interview with Author, 2005.

97 The US has been an unparalleled leader in funding the global response to AIDS through PEPFAR and other programmes, but its approach has also been criticised for failing to negotiate the best possible drug prices; for refusing to fund health care infrastructures critical to the delivery of medication; and for exporting US ideologies about sex, abstinence and faithfulness into contexts where they constrain and undermine the efforts of public health workers. See, for example, Anne Esacove, ‘Love Matches: Heteronormativity, Modernity, and AIDS Prevention in Malawi’,’ *Gender & Society*, 2010, 24, 83–109.

98 Presidents George H. W. Bush and George W. Bush both took careful guidance on HIV/AIDS from Anthony Fauci, a ground-breaking AIDS researcher and long-time Director of the National Institute of Allergy and Infectious Diseases (NIAID). In this and other respects they distinguished themselves from prominent conservatives who refused to lead on the issue. John Donnelly, ‘The President’s Emergency Plan for AIDS Relief: How George W. Bush and Aides Came to “Think Big” on Battling HIV’, *Health Affairs (Project Hope)*, 2012, 31, 1389–96, doi:10.1377/hlthaff.2012.0408; Scott Evertz, ‘How Ideology Trumped Science: Why PEPFAR Has Failed to Meet Its Potential’ (Center for American Progress, January 2010), <https://www.americanprogress.org/issues/healthcare/reports/2010/01/13/7214/how-ideology-trumped-science/>. The Bush administration so thoroughly reframed AIDS as a global issue that, in the 2004 debates, both vice presidential candidates found themselves unprepared to answer a question about domestic HIV/AIDS policy and quickly diverted their responses to the African context. Commission on Presidential Debates, ‘The Cheney–Edwards Vice Presidential Debate, October 5 Transcript’, *Commission on Presidential Debates*, 2004, <http://www.debates.org/index.php?page=october-5-2004-transcript>.

99 James A. Morone, *Hellfire Nation: The Politics of Sin in American History* (New Haven, CT: Yale University Press, 2003); Padamsee, ‘The Politics of Prevention: Lessons from the Neglected History of U.S. HIV/AIDS Policy’, 2017.

100 On the Surgeon General’s advice, see Kaiser Family Foundation, ‘Kaiser Daily HIV/AIDS Report’, 2001, 2002, and 2003.

101 Jonathan Saltzman, ‘ACLU Sees Religious Content in Abstinence Plan’, *The Boston Globe*, 17 May 2005, sec. Education; American Foundation for AIDS Research (AMFAR), ‘Assessing the Efficacy of Abstinence-Only Programs for HIV Prevention Among Young People’, Issue Brief (Washington, DC: American Foundation for AIDS Research, April 2005); Tamar Lewin, ‘Quick Response to Study of Abstinence Education’, *New York Times*, 3 February 2010, sec. A18; Harold Jaffe, Interview with Author, 2011; Kaiser Family Foundation, ‘Kaiser Daily HIV/AIDS Report’, 2002.

102 Kaiser Family Foundation, ‘Kaiser Daily HIV/AIDS Report’, 2002.

103 Kaiser Family Foundation, ‘Kaiser Daily HIV/AIDS Report’, 2004 and 2005.

104 Mireya Navarro, ‘Experts in Sex Field Say Conservatives Interfere with Health and Research’, *The New York Times*, 11 July 2004, sec. 116; Kaiser Family Foundation, ‘Kaiser Daily HIV/AIDS Report’, 2001 and 2002.

105 Confidential Informant #12, Interview with Author, 2005.

106 Kaiser Family Foundation, ‘Kaiser Daily HIV/AIDS Report’, 2002 and 2003.

107 Congressional pressure to curtail funding of peer-reviewed research that legislators deem unimportant or inappropriate has since continued, and academic organisations continue to resist these limits on academic freedom and advocate for increased investment in health research. For example, the Union of Concerned Scientists has mounted a Scientific Integrity project to monitor and protect health, safety, and environmental research from political interference. Confidential Informant #20, Interview with Author, 2005; Kaiser Family Foundation, ‘Kaiser Daily HIV/AIDS Report’, 2003; Research!America, ‘Research!America: An Alliance for Discoveries in Health’, 2015, <http://www.researchamerica.org/>; Union of Concerned Scientists, ‘Scientific Integrity’, n.d., <http://www.ucsusa.org/scientific_integrity/>.

108 Jeffrey Levi, ‘An HIV Agenda for the New Administration’, *American Journal of Public Health*, 2001, 91, 1015–16; Confidential Informant #33, Interview with Author. While SAFE funnelled much-needed funds to organizations working with infected individuals, it also diverted significant resources from prevention programmes for youth in high-risk communities. Chris Norwood, ‘Positively Ignored’, *The New York Times*, 5 December 2004, sec. 14WC19; David Harvey, Interview with Author, 2005; Kaiser Family Foundation, ‘Kaiser Daily HIV/AIDS Report’, 2004; Confidential Informant #24, Interview with Author.

109 Bernard Branson *et al.*, ‘Revised Recommendations for HIV Testing of Adults, Adolescents, and Pregnant Women in Health-Care Settings’, *Morbidity and Mortality Weekly Report* 55, no. RR14 (2006), 1–17.

110 Bernard Branson, Interview with Author, 2011.

111 In addition to the positive impacts of routinising testing policies, there is some evidence of unintended negative consequences, including use of these policies to justify coercive testing of some marginalised groups in the US South. Harvey, Interview with Author; Bronwen Lichtenstein, Interview with Author, 2011; Padamsee, ‘The Politics of Prevention: Lessons from the Neglected History of U.S. HIV/AIDS Policy’, 2017; Richard Perez-Pena, ‘New Federal Health Policy on HIV Testing Poses Unique Local Challenge’, *The New York Times*, 2 October 2006, sec. B1.

112 ‘Proposal Calls For Medicare Coverage Of HIV Screening’, *Kaiser Health News*, 10 September 10, 2009, <http://khn.org/morning-breakout/medicare-and-hiv/>; ‘Preventive Services Task Force Urges HIV Screening For Adults’, *Kaiser Health News*, 30 April 2013, <http://khn.org/morning-breakout/hiv-issues/>; ‘Panel Backs Routine HIV Screenings For Teens, Adults’, *Kaiser Health News*, 20 November 2012, <http://khn.org/morning-breakout/hiv-testing/>; ‘Taking HIV Testing to the Streets | KQED’, *The California Report*, 15 September 2011, <http://audio.californiareport.org/archive/R201109150850/b>; ‘Medicare To Pay For HIV Testing’, *Kaiser Health News*, 9 December 2009, <http://khn.org/morning-breakout/medicare-hiv-screening/>; Amanda Waldroupe, ‘Oregon House and Senate Unanimously Agree on New Changes to HIV Testing | The Lund Report’, 22 February 2012, <https://www.thelundreport.org/content/oregon-house-and-senate-unanimously-agree-new-changes-hiv-testing>; ‘HIV Testing for All’, *Los Angeles Times*, 23 November 2012, <http://articles.latimes.com/2012/nov/23/opinion/la-ed-hiv-test-20121123>; Julie Steenhuysen, ‘Task Force Calls for Routine HIV Testing for All Adults’, *Reuters*, 30 April 2013, <http://www.reuters.com/article/us-usa-hiv-testing-idUSBRE93S0XZ20130430>; Lichtenstein, Interview with Author.

113 Shaila Dewan, ‘In Mississippi, Soaring Costs Force Deep Medicaid Cuts’, *New York Times*, 2 July 2005, sec. A; Robert Pear, ‘U.S. Gives Florida a Sweeping Right to Curb Medicaid’, *New York Times*, 20 October 2005, sec. A; Robert Pear, ‘New Medicaid Rules Allow States to Set Premiums and Higher Co-Payments’, *New York Times*, 27 November 2008, sec. A; Kaiser Family Foundation, ‘Kaiser Daily HIV/AIDS Report’, 2005 and 2006.

114 Confidential Informant #3, Interview with Author, 2005; Confidential Informant #5, Interview with Author, 2005; Confidential Informant #30, Interview with Author.

115 ‘AIDS Money Suddenly At Risk’, *New York Times*, 1 September 2006, sec. A16; Erik Eckholm, ‘H.I.V. Patients Anxious as Support Programs Cut Back’, *New York Times*, 1 August 2007, sec. A12; Gebbie, Interview with Author; Gary Rose, Interview with Author, 2005; Confidential Informant #12, Interview with Author; Confidential Informant #13, Interview with Author, 2005.

116 Julia Preston, ‘Obama Lifts a Ban on Entry into the U.S. by H.I.V.-Positive People’, *The New York Times*, 31 October 2009, sec. A.

117 On the signing of the CARE Act, see Bernie Becker, ‘AIDS Funding Bill Sails through the House’, *New York Times*, 21 October 2009. For the adminstration’s public meetings, see Confidential Informant #5, Interview with Author, 2011.

118 SIECUS, ‘A Brief History of Federal Funding for Sex Education and Related Programs’, *Sexuality Information and Education Council of the United States*, 2010, <http://www.siecus.org/index.cfm?fuseaction=page.viewpage&pageid=1341&nodeid=1> (accessed on 15 December, 2015).

119 Confidential Informant #102, Interview with Author, 2010; Confidential Informant #105, Interview with Author, 2011.

120 The Washington Times, ‘Supreme Court Strikes down Obama-Backed “Prostitution Pledge” in AIDS Funding’, *The Washington Times*, 20 June 2013, <https://www.washingtontimes.com/news/2013/jun/20/supreme-court-nixes-prostitution-pledge-aids-funds/>.

121 ‘Righting a Wrong, Much Too Late’, *New York Times*, 26 December 2009, sec. A22; Bob Egelko, ‘U.S. Repeals Funding Ban for Needle Exchanges’, *San Francisco Chronicle*, 18 December 2009; Katie Zezima, ‘Provision in a Bill Aimed at Helping Needle Exchanges Would Hurt Most of Them’, *New York Times*, 9 November 2009, sec. A9; Preston, ‘Obama Lifts a Ban on Entry into the U.S. by H.I.V.-Positive People’; Confidential Informant #157, Interview with Author, 2011; Kaiser Family Foundation, ‘Kaiser Daily HIV/AIDS Report’, 2009; Kaiser Family Foundation, ‘Kaiser Health News Daily Health Policy Report’, 2010, <http://www.kaiserhealthnews.org/Headlines.aspx>; POZ Magazine, ‘POZ News Top Stories’, 2009, <http://www.poz.com/archive/currentNews_1.shtml>; POZ Magazine, ‘POZ News Top Stories’, 2010, <http://www.poz.com/archive/currentNews_1.shtml>.

122 Confidential Informant #105, Interview with Author.

123 Sarah Barr, ‘Needle Exchange Programs Face New Federal Funding Ban’, *Kaiser Health News*, 21 December 2011, <http://www.kaiserhealthnews.org/stories/2011/december/21/needle-exchange-federal-funding.aspx>.

124 AVERT, ‘Needle Exchange and Harm Reduction’, n.d., <www.avert.org/needle-exchange.htm>; Vivian Guardino *et al.*, ‘Syringe Exchange Programs—United States, 2008’, Morbidity and Mortality Weekly Report, 2010; Mathers *et al.*, ‘Global Epidemiology of Injecting Drug Use and HIV among Peole Who Inject Drugs: A Systematic Review’; NASEN, ‘U.S. Syringe Exchange Database’ (North American Syringe Exchange Network, n.d.), <https://nasen.org/map/>; Laura Whitehorn, ‘The War on Drugs Spreads HIV’, *POZ*, November 2012<https://www.poz.com/article/global-drug-war-22984-9082>.

125 ‘Preventing HIV and Hepatitis Among People Who Inject Drugs and Their Partners’ (AIDS.gov, 20 January 2017), <https://www.hiv.gov/federal-response/policies-issues/syringe-services-programs>; Michael Botticelli, ‘Bipartisan Budget Agreement Builds on President’s Call for New Funding in Opioid Epidemic’, *White House Blog*, 31 December 2015, <https://www.whitehouse.gov/blog/2015/12/30/more-funding-opioid-epidemic-bipartisan-budget-agreement>.

126 Confidential Informant #102, Interview with Author; Confidential Informant #5, Interview with Author, 2011. ONAP articulated a National Strategy in 1997 United States., *The National AIDS Strategy, 1997: President Clinton, August 29, 1996, Chicago, Illinois.*, 40 p. ([Washington, DC: U.S. White House Office] : For sale by the U.S. G.P.O., Supt. of Docs., 1997), <https://catalog.hathitrust.org/Record/003178488>, under Clinton, but took a quiet, behind-closed-doors approach that HIV/AIDS advocates blamed for the Strategy’s limited effectiveness. As an experienced policy researcher and former leader of the National Association of People with AIDS (NAPWA), Crowley was well aware of the history; this time, therefore, ONAP convened 14 large meetings across the country with HIV/AIDS activists, organisation leaders, grantees and members of the general public, and it conducted in-depth information gathering exercises with several government agencies.

127 Office of National AIDS Policy, ‘National HIV/AIDS Strategy: Federal Implementation Plan’, 2010, <https://files.hiv.gov/s3fs-public/nhas-implementation.pdf>.

128 Timothy Westmoreland, Interview with Author, 2011; Baker, Interview with Author.

129 Robert Pear, ‘Obama to Outline Plan to Cut H.I.V. Infections’, *New York Times*, 11 July 2010.

130 Crews, Interview with Author; Scott Evertz, Interview with Author, 2011; Sam Friedman, Interview with Author, 2011; Holtgrave, Interview with Author; Levi, Interview with Author, 2011; Confidential Informant #3, Interview with Author, 2011; Confidential Informant #6, Interview with Author, 2011.

131 Department of Health and Human Services, ‘National HIV/AIDS Strategy: Implementation Progress Report 2012’ (Department of Health and Human Services, 2013), <https://files.hiv.gov/s3fs-public/hhs-2012-nhas-progress-report.pdf>; Confidential Informant #20, Interview with Author, 2011; Crews, Interview with Author; Confidential Informant #5, Interview with Author, 2011; Project Inform, ‘Frequently Asked Questions about Health Care Reform for People Living with HIV’, *Project Inform: Information, Inspiration and Advocacy for People with HIV/AIDS and Hepatitis C*, 2011, <http://www.projectinform.org/publications/frequently-asked-questions-about-health-care-reform/>; Javier Salazar, Interview with Author, 2011; Murray Penner and Jen Kates, ‘AIDS Drug Assistance Programs: A Lifeline for People with HIV’ (The Body: the Complete HIV/AIDS Resource, 2008), <http://www.thebody.com/content/art52441.html#8>; Confidential Informant #13, Interview with Author, 2011; Robbie Brown, ‘Ruling Soon on Isolation of Inmates With H.I.V.’, *The New York Times*, 19 October 2012, <http://www.nytimes.com/2012/11/19/us/isolation-of-hiv-inmates-in-alabama-at-issue.html>; Robbie Brown, ‘Alabama to End Isolation of Inmates With H.I.V.’, *The New York Times*, 22 December 2012, sec. A, <http://www.nytimes.com/2012/12/22/us/alabama-to-end-isolation-of-inmates-with-hiv.html?_r=0>; Verna Gates, ‘Alabama’s Segregation for Inmates with HIV Faces Court Scrutiny’, *Reuters*, 17 September 2012, <http://www.reuters.com/article/2012/09/17/us-usa-alabama-hiv-idUSBRE88G0KS20120917>.

132 Project Inform, ‘Frequently Asked Questions about Health Care Reform for People Living with HIV’; Salazar, Interview with Author; Confidential Informant #3, Interview with Author, 2011.

133 Penner and Kates, ‘AIDS Drug Assistance Programs: A Lifeline for People with HIV’; Confidential Informant #13, Interview with Author, 2011.

134 Even before it was fully implemented, the ACA helped move many HIV-positive people onto insurance and reduce ADAP waiting lists through its requirement that insurance plans cover individuals with pre-existing conditions. It remains to be seen how Trump-era enthusiasm for dismantling the ACA and related health care laws will impact insurance coverage for HIV-positive people, or their access to options such as PrEP through insurance programmes.

135 Other causal factors are also undoubtedly important, including long-time activist efforts to cope with stigma, scientific and treatment advancements that have made HIV less threatening and a political environment (e.g. the increasing social acceptance of gay rights) relatively friendly to such changes. Brown, ‘Ruling Soon on Isolation of Inmates With H.I.V.’; Brown, ‘Alabama to End Isolation of Inmates With H.I.V’; Gates, ‘Alabama’s Segregation for Inmates with HIV Faces Court Scrutiny’; Christina Gonzalez, ‘Condoms Could Soon Be Legally Distributed in US Prisons’, *POZ*, December 2011; Cynthia Gordy, ‘Notorious HIV: The Criminal Prosecution of a Virus’, *The Root*, 11 March 2011, <https://www.theroot.com/notorious-hiv-the-criminal-prosecution-of-a-virus-1790863108>; Jacqueline Mroz, ‘Gay Men Condemn Blood Ban as Biased’, *The New York Times*, 3 August 2010; Trenton Straube, ‘Bar None’, *POZ*, May 2013; Katie Thomas, ‘Equestrian Charged With H.I.V.-Related Offense’, *The New York Times*, 12 April 2010, sec. A; Reed Vreeland, ‘Tainted Blood Policy’, *POZ*, December 2011; Lichtenstein, Interview with Author; Salazar, Interview with Author; Scofield, Interview with Author, 2011; Matthew Weait, Interview with Author, 2011; Confidential Informant #8, Interview with Author; Confidential Informant #17, Interview with Author, 2011; Confidential Informant #112, Interview with Author, 2011; Confidential Informant #157, Interview with Author; Kaiser Family Foundation, ‘Kaiser Daily HIV/AIDS Report’, 2007, Kaiser Family Foundation, ‘Kaiser Health News Daily Health Policy Report’, 2010; POZ Magazine, ‘POZ News Top Stories’, 2010; POZ Magazine, ‘POZ News Top Stories’, 2011, <http://www.poz.com/archive/currentNews_1.shtml>; POZ Magazine, ‘POZ News Top Stories’, 2012, <http://www.poz.com/archive/currentNews_1.shtml>; Elton John, ‘HIV-Positive Prisoners in Alabama and South Carolina Face Discriminatory Practices’, *The Washington Post*, 16 September 2012, <https://www.washingtonpost.com/opinions/elton-john-hiv-positive-prisoners-in-alabama-and-south-carolina-face-discriminatory-practices/2012/09/16/439754bc-feaf-11e1-b153-218509a954e1_story.html?utm_term=.e20cf7ccf92d>; Adrienne Lu, ‘Filling of Vacancies Lags in County Health System’, *The New York Times*, 10 October 2011, <http://www.nytimes.com/2011/11/11/us/filling-of-vacancies-lags-in-county-health-system.html>.

136 Salazar, Interview with Author.

137 Laura Whitehorn, ‘More Access to Medicaid for People With HIV’, *POZ*, September 2011; ‘Many People on Therapy for HIV Should Qualify for Life Insurance’, *POZ*, 1 April 2013; Kaiser Family Foundation, ‘Kaiser Health News Daily Health Policy Report’, 2013, <http://www.kaiserhealthnews.org/Headlines.aspx>.

138 Centers for Disease Control and Prevention, ‘HIV in the United States: At a Glance’.

139 The specific groups being referenced here are: African Americans, women, Native Americans and those living in the US South (the most heavily affected region). Baker, Interview with Author; Evertz, Interview with Author; Kathie Hiers, Interview with Author, 2011; Carl Schmid, Interview with Author, 2011; White House Office of National AIDS Policy, ‘National HIV/AIDS Strategy for the United States’.

140 Rental assistance for unstably housed and homeless persons living with HIV has been shown to reduce HIV viral load, emergency room use and perceived stress and to provide considerable cost savings. David R. Holtgrave *et al.*, ‘Cost–Utility Analysis of the Housing and Health Intervention for Homeless and Unstably Housed Persons Living with HIV’, *AIDS and Behavior*, 2013, 17, 1626–31; Hiers, Interview with Author; Holtgrave, Interview with Author.

141 HIV.gov, ‘What is the HIV Care Continuum?’, 6 March 2015, <https://www.hiv.gov/federal-response/policies-issues/hiv-aids-care-continuum>; Confidential Informant #29, Interview with Author, 2011; Hiers, Interview with Author; Lichtenstein, Interview with Author; Confidential Informant #21, Interview with Author, 2011.

142 Although this history does not extend to the policy actions of the Trump administration, the unfolding story during the first year of the shift back to national-level Republican control suggests a swing back in the direction seen under prior Republican administrations. Domestic HIV/AIDS policy is no longer a subject of substantial discussion, the Department of Health and Human Services’ focus on religious freedom for insurers may render prevention options such as PrEP less frequently available, and the end of the individual tax penalty for not carrying health insurance reflects Republican leaders’ priority on curtailing as many of the ACA’s access-expanding provisions as possible in lieu of repealing the ACA altogether. Department of Health and Human Services, ‘HHS Announces New Conscience and Religious Freedom Division’, *HHS Press Release*, 18 January 2018, <https://www.hhs.gov/about/news/2018/01/18/hhs-ocr-announces-new-conscience-and-religious-freedom-division.html>; Robert Pear, ‘Individual Mandate Now Gone, G.O.P. Targets the One for Employers’, *New York Times*, 14 January 2018, <https://www.nytimes.com/2018/01/14/us/politics/employer-mandate.html?login=email&auth=login-email>.

